# Macrophage polarization state influences fusion and phenotype of human foreign body giant cells *in vitro*

**DOI:** 10.3389/fimmu.2026.1832686

**Published:** 2026-07-21

**Authors:** Thijs S. Conner, Bente J. de Kort, Anthal I. P. M. Smits

**Affiliations:** 1 Department of Biomedical Engineering, Eindhoven University of Technology, Eindhoven, Netherlands; 2 Institute for Complex Molecular Systems (ICMS), Eindhoven University of Technology, Eindhoven, Netherlands

**Keywords:** biomaterials, cell fusion, foreign body response, immune response, inflammation, interleukin-4, macrophage plasticity, multinucleated giant cells

## Abstract

**Introduction:**

Macrophages and their fused counterpart, foreign body giant cells (FBGCs), have a determining influence on the long-term functionality of implanted biomaterials, coordinating biomaterial resorption and fibrous encapsulation. Modulating macrophage phenotype into favorable polarization states is a widely adopted strategy to attempt steering the foreign body response. However, the links between macrophage phenotype and FBGC formation and function are unknown. The goal of this study was to determine the fusion potential of well-defined macrophage polarization states, and to explore whether prior polarization state translates to FBGC phenotype in primary human macrophages.

**Methods:**

Peripheral blood-derived human macrophages were first stimulated toward M0/M1/M2a/M2c polarizations after which they were stimulated to fuse into FBGCs using interleukin-4 (IL-4). After fusion, these cells were re-stimulated to investigate the effect of the respective cytokine cocktails on FBGC phenotype.

**Results:**

The results showed that M1 macrophages had increased IL-4 receptor expression, an altered cytoskeleton, and significantly increased expression of fusion-associated genes, when compared to unstimulated, M2a and M2c macrophages. FBGCs could form from all polarization states. Although M1 polarization led to a decreased fusion percentage, the FBGCs that did form were larger in terms of cell size and number of nuclei. FBGCs showed significant changes in their cytoskeletal structure by forming rings of actin around the periphery of the cell, which was consistent for the different macrophage polarizations. Expression of select genes and proteins associated with macrophage polarization states were either maintained during fusion, or reacquired by FBGCs upon restimulation with polarizing factors.

**Discussion:**

These results provide evidence for the existence of FBGC polarization states, emphasizing the importance of the macrophage population and microenvironment in regulating FBGC formation. This suggests that FBGC function may be steerable, for example using biomaterial design parameters, in order to improve long-term biomaterial functionality and integration.

## 1 Introduction

The host response to a foreign body, such as an implanted biomaterial, is a cascade of events that is triggered by tissue injury. Following acute inflammation, which is dominated by neutrophils, macrophages are recruited to the inflamed area, marking the onset of chronic inflammation (1–3). During this phase, macrophages regulate tissue restoration depending on their polarization state, in cross-talk with (myo)fibroblasts and T-cells (2, 4). Macrophage phenotypes and functions are diverse and plastic and depend on the local environmental cues, such as activation signals that are sensed via pattern recognition receptors and podosomes, in combination with cytokines (5–7). For example, the presence of T-helper 1 (Th1)-associated cytokines, such as interferon gamma (IFN-γ), and/or damage- or pathogen-associated molecular patterns (e.g. lipopolysaccharide, LPS), leads to pro-inflammatory macrophage polarization (7–9). Such pro-inflammatory macrophages sustain inflammation via secretion of pro-inflammatory cytokines, as well as degradative species, such as hydrolytic enzymes and reactive oxygen species (ROS) (10, 11). Especially through these degradative species, pro-inflammatory macrophages can participate in biomaterial degradation. Alternatively, when exposed to Th2/T_reg_-associated cytokines, such as interleukin-4 (IL-4), IL-13 or IL-10, macrophages can attain a range of anti-inflammatory phenotypes, which stimulate tissue formation and remodeling via the secretion of factors, such as platelet derived growth factor, matrix and metalloproteinases (MMPs), as well as immune regulation (e.g. via IL-10) (12–14).

If the foreign body persists in the host, this typically leads to the foreign body response (FBR). The FBR is characterized by fibrous encapsulation and/or the fusion of macrophages into foreign body giant cells (FBGCs). Previous research has shown that macrophages start fusing into FBGCs to more efficiently phagocytose or degrade foreign bodies larger than approximately 10 µm in size (15). Research by Kreipe et al. showed that the production of oxygen free radicals in response to zymosan was 20–30 fold increased in FBGCs when compared to unfused macrophages (16). Likewise, Matheson et al. showed that few FBGCs were more efficient in eliciting degradation when compared to a greater number of mononucleated macrophages (17). Additionally, the presence of FBGCs on non-degradable biomaterials is associated with fibrous encapsulation and myofibroblast activation (18). As such, the FBR in response to an implanted biomaterial can lead to detrimental events, such as excess fibrosis and tissue contraction, which may critically impair functionality of implants (19–22). However, when harnessed properly, the host response to an implant can also be used to induce tissue regeneration, for example by using resorbable biomaterials (i.e. *in situ* tissue engineering) (23, 24).

Macrophage fusion into FBGCs is a multistage process that involves adhesion of macrophages to a substrate, the induction of a fusion-competent state, juxtacrine cell-cell interactions, cytoskeletal rearrangements, and subsequent membrane fusion to acquire multinucleation (25). Interestingly, the cytokines IL-4 and IL-13, as secreted by Th2 cells *in vivo* and which are known inducers of a pro-fibrogenic macrophage polarization state, have been pinpointed as dominant stimuli for acquiring fusion-competent macrophages (26). For both IL-4 and IL-13 the signaling starts upon binding to the IL-4/IL-13 receptor complex of the macrophage, which upregulates known fusogens, including dendritic cell-specific transmembrane protein (DC-STAMP) and E-Cadherin (27, 28), through the Signal Transducer And Activator Of Transcription 6 (STAT6) and STAT1 axes (28, 29). Another route to gain a fusion-competent state in macrophages is through DNAX-activating protein 12 (DAP12) signaling, for which activation has been implied with cell-cell contact (30, 31). When in close contact, the fusion-competent macrophage must recognize the cell membrane of another macrophage. This is initiated by actin-rich protrusions originating from a podosome-rich region (32). Upon macrophage recognition, adhesion is started by formation of a zipper-like structure between the two cell membranes and additionally an actin-rich structure is formed on the interior side of both cell membranes (33). Lastly, the cell membranes fuse together, forming a pore to share the cytoplasm and nuclei, and rearrange their cytoskeletal structures to ensure the functioning of the now multinucleated cell.

Numerous preclinical studies have shown the importance of both macrophages and FBGCs in the *in situ* regeneration of blood vessels and heart valves based on resorbable biomaterials (34–40). The presence of FBGCs and macrophages was associated with local resorption of the biomaterial and the formation of new tissue, suggesting that both macrophages and FBGCs play a key role in steering the balance between biomaterial degradation and neo-tissue formation and remodeling, which is essential for sustained implant functionality (34). Moreover, it was shown that early macrophage polarization state was correlated with long-term outcome of an implant, particularly for natural biomaterials that can be degraded or remodeled relatively efficiently (41–43). Given the latter, steering macrophage polarization state using biomaterials has become a target for inducing a favorable host response to implants. Biomaterial-induced macrophage polarization, for example via IL-4 functionalization and/or release, has become a broadly adopted strategy aimed to improve implant integration or tissue regeneration *in situ* (43–50). It is, however, not known how macrophage polarization states relate to fusion potential into FBGCs, and whether or not macrophage phenotype prior to fusion affects FBGC function. The different functions of FBGCs have led to the proposition that FBGCs may share a phenotypic heterogeneity equivalent to that of macrophages. The goal of this study is to investigate how macrophage polarization state affects fusion potential, and to what extent macrophage polarization state prior to fusion, affects FBGC phenotype.

We hypothesize that IL-4-induced FBGC formation is mainly driven from pro-inflammatory macrophages, as previous research in zebrafish has shown that FBGCs formed in a pro-inflammatory environment during the FBR (51). Furthermore, it has been shown that LPS/IFN-γ-induced macrophages expressed higher IL-4 receptor alpha (IL-4Rα) expression than their unprimed and IL-4-induced counterparts (52). To test this hypothesis, we examined FBGC formation from primary human macrophages that were either unprimed (‘M0’), or stimulated with LPS/IFN-γ (‘M1’), IL-4 (‘M2a’), or IL-10 (‘M2c’) polarizing agents, prior to fusion. Furthermore, we investigated if the formed FBGCs showed similar polarization characteristics as macrophages in response to macrophage polarization cocktails.

## 2 Materials and methods

### 2.1 Establishing macrophage fusion protocols

No standardized protocols exist for culturing primary human FBGCs, as recently reported (47). Therefore, two specific experimental protocols were developed and used to test our hypothesis (**Figure 1**). In brief, we first developed a fusion protocol for primary human macrophages (Protocol A) and established the time course of fusion. Based on the results, we established a second fusion protocol, which is less sensitive to spontaneous macrophage fusion and donor-to-donor variability, prior to IL-4 stimulation (Protocol B). For protocol A, primary human cluster of differentiation 14 positive (CD14+) monocytes were isolated from buffy coats using negative selection with magnetic-activated cell sorting (MACS). The monocytes were seeded directly in well plates in MEMα medium with macrophage colony stimulating factor (M-CSF) for 5 days to induce monocyte-to-macrophage differentiation. Subsequently, macrophage polarization was induced by stimulating with LPS/IFN-γ (termed ‘M1’ throughout the study), IL-4/IL-13 (‘M2a’) or IL-10 (‘M2c’). After 48 hours, the polarization stimuli were removed, and macrophage fusion was induced by addition of 50 ng/ml IL-4. For protocol B, macrophages were first cultured in RPMI-1640 medium with M-CSF in culture flasks, as we found that this medium does not support fusion, but does support polarization similarly to MEMα (**Supplementary Figure 1**). After 5 days of culture, macrophages were detached and macrophages of 6–9 different donors were pooled together. The pooling of macrophages served three main purposes: (1) to reduce donor-to-donor variability, (2) to reduce costs by omitting MACS isolation, and (3) to increase the number of readouts that can be performed per donor, as this removes the limit of total number of cells of just one donor. The pooled cells were seeded in well plates and subsequently polarized for 48 hours in RPMI-1640, followed by induction of fusion with IL-4 in MEMα medium at an optimized concentration of 10 ng/ml. Full details are described in the following sections.

**Figure 1:**
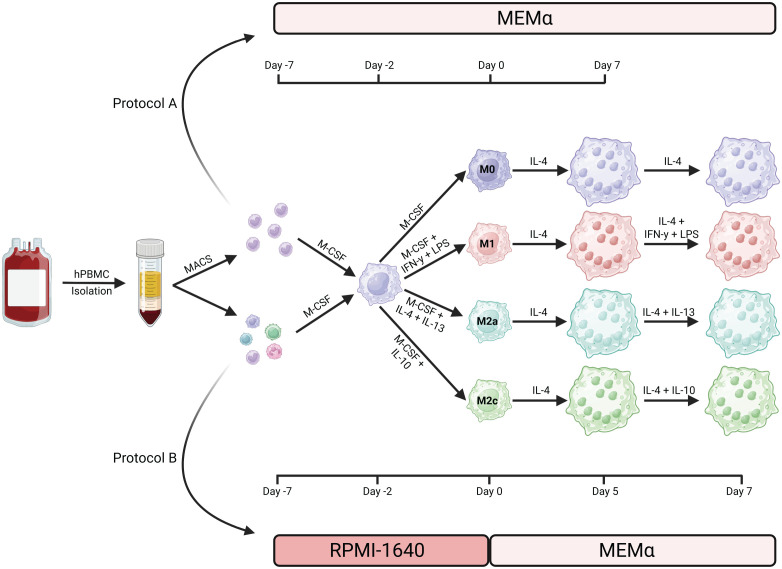
Experimental overview. Human peripheral blood mononuclear cells (hPBMCs) were isolated from buffy coats using density gradient centrifugation. Afterwards, hPBMCs were either directly seeded in culture flasks or cluster of differentiation 14 positive monocytes were isolated using magnetic assisted cell sorting (MACS). hPBMCs or isolated monocytes were then differentiated toward macrophages using macrophage colony stimulating factor (M-CSF) for 5 days (Day -7 to Day -2). Afterwards, macrophages were polarized into three different polarization states (M1; using interferon-gamma (IFN-γ) and lipopolysaccharide (LPS), M2a; using Interleukin-4 (IL-4) and IL-13, and M2c; using IL-10), or kept unpolarized by only providing M-CSF (M0) for two days (Day -2 to Day 0). hPBMCs were cultured in RPMI-1640, while MEMα was used for isolated monocytes. Fusion of macrophages into foreign body giant cells (FBGCs) was induced by addition of IL-4 for 5/7 days in MEMα medium (Day 0 to Day 5 or 7). After FBGC formation, polarization stimuli were added to the FBGCs for 2 days (Day 5 to Day 7). Created in BioRender. Conner, T. (2026) https://BioRender.com/gesc88t.

### 2.2 General cell culture components

Two different types of basal medium were used in this research, RPMI-1640 (A1049101; Gibco) and MEMα (41061; Gibco). Both media were supplemented with 5% human platelet lysate (PLBioScience) and penicillin/streptomycin (1% v/v, Lonza) and will be termed as “Complete RPMI-1640/MEMα” unless otherwise stated. All cytokines used were obtained from Peprotech. Unless otherwise stated cells were cultured in 24 well plates (Greiner) with 13 mm Ø coverslips (VWR).

### 2.3 Human peripheral blood mononuclear cell isolation and macrophage differentiation

Human buffy coats from healthy anonymized donors (details in **Table 1**) were purchased from Sanquin blood supply foundation, Nijmegen, the Netherlands, under informed consent from the donors and with approval by the ethical committee of Sanquin. A 0.6% (w/v) sodium citrate (Merck) in phosphate buffered saline (PBS; ThermoFisher) was used to dilute the buffy coat, after which isolation of human peripheral blood mononuclear cells (hPBMCs) was performed using density gradient centrifugation on iso-osmotic medium (lymphoprep, StemCell). Isolated hPBMCs were frozen at -150 °C in RPMI-1640 medium with 20% fetal bovine serum (Serana) and 10% dimethyl sulfoxide (Sigma-Aldrich). When needed, cells were thawed and washed using pre-warmed complete RPMI-1640 medium. For protocol A, CD14+ monocytes were isolated from fresh (never frozen) hPBMCs using negative selection MACS (Miltenyi Biotec) according to the manufacturer’s protocol. The monocytes were seeded at a density of 2*10^5^ cells/cm^2^ and cultured for 5 days with 50 ng/ml M-CSF in complete MEMα. To induce polarization, different cytokine cocktails were added alongside 50 ng/ml M-CSF. M0 macrophages did not receive any additional stimulant. M1 macrophages were provided with 20 ng/ml LPS and 20 ng/ml IFN-γ. IL-4 and IL-13 were added for M2a polarization at 40 and 20 ng/ml respectively. Lastly, M2c polarization was induced by adding 20 ng/ml IL-10.

**Table 1:** Known patient characteristics of used donors.

Donor #	Age (y)	Sex	Blood type	Pool
399	27	Female	A+	1
087	64	Female	A+	1
344	24	Female	A+	1
917	37	Female	A+	1
332	44	Female	A+	1
052	36	Male	A+	1
338	28	Female	A+	2
390	23	Female	A+	2
831	65	Female	O+	2
839	37	Female	O+	2
837	39	Female	B+	2
835	75	Male	O+	2
109	63	Female	B+	2
835	75	Male	O+	3
390	23	Female	A+	3
917	37	Female	A+	3
052	36	Male	A+	3
087	64	Female	A+	3
399	27	Female	A+	3
344	25	Female	A+	3
332	44	Female	A+	3
837	39	Female	B+	3
293	72	Male	B+	4
503	71	Male	B+	4
120	75	Male	A+	4
476	40	Female	A+	4
561	71	Male	A+	4
939	29	Female	A+	4
610	71	Male	A+	4
837	39	Female	B+	4
694	25	Female	A+	N.A.
699	51	Male	A+	N.A.
708	60	Male	O+	N.A.
503	58	Male	A+	N.A.
501	20	Female	A+	N.A.
400	65	Male	A+	N.A.
432	53	Female	A+	N.A.
899	55	Male	A+	N.A.
420	26	Male	A+	N.A.
742	72	Male	O+	N.A.
412	75	Male	A+	N.A.
769	21	Female	A+	N.A.
136	55	Female	A+	N.A.

For protocol B, after thawing, hPBMCs were resuspended in complete RPMI-1640 containing 50 ng/ml macrophage-colony stimulating factor. 50*10^6^ hPBMCs were seeded in a T75 suspension culture flask (Cellstar^®^; Greiner) and cultured for 5 days with a medium refresh after 2–3 days. Afterwards, attached cells were deemed as macrophages and were detached using Accutase (Stemcell) for 10–30 minutes at 37 °C. Macrophages of 6–9 donors were then pooled together with an equal number of macrophages per donor (**Table 1**, Pool 1-4). Subsequently, macrophages were seeded at 125-150*10^3^ cells/cm^2^ in complete RPMI-1640 with their specific polarization cocktail. The polarization cocktail added for protocol B was similar to that from protocol A except for M1 polarization where the concentration for both LPS and IFN-γ was 100 ng/ml instead of 40 and 20 ng/ml, respectively, to always ensure sufficient polarization.

For both protocols, after 2 days of polarization cells were either sacrificed for analysis, or medium was switched to complete MEMα medium in combination with 50 ng/ml IL-4 for protocol A or 10 ng/ml IL-4 for protocol B to induce fusion into FBGCs. Medium was refreshed every 2–3 days for a total of 5 days of fusion induction. Obtained cells were then either sacrificed for analysis or were further polarized. To induce polarization of FBGCs, each group (i.e. M0, M1, M2a, M2c) received IL-4 in complete MEMα combined with the cytokine cocktail as detailed above, with the exception that no M-CSF was added in any condition. After 2 days of polarization, cells were sacrificed for analysis.

### 2.4 Immunofluorescence imaging readouts

Cells were fixated for 10–30 minutes in 3.7% formaldehyde (Sigma) and washed with PBS. Afterwards, cells were permeabilized using 0.5% Triton-x-100 (Merck) and washed with 0.5% PBS-tween (PBS-T; Merck). Samples were then either stained using phalloidin (Sigma-Aldrich) and DAPI (1:200; Sigma-Aldrich) for 30 minutes or using the following protocol for antibody stainings: to reduce non-specific staining, samples were blocked using 5% goat serum and 1% bovine serum albumin (BSA; Merck) in 0.5% PBS-T. Primary antibodies were diluted in 10x diluted blocking solution (**Table 2**) and incubated with the cells either overnight at 4 °C or for 1.5h at room temperature. After washing with PBS-T, the secondary antibodies were incubated for 1h at room temperature. During this step phalloidin was also added. Lastly, after washing, samples were stained with DAPI for 5 minutes at room temperature. For both staining procedures, samples were then washed with PBS and coverslips were mounted on microscopy slides using Mowiol. Imaging was performed using the Zeiss observer 7 (Carl-Zeiss) or DMI8 (Leica). For each sample, 5–10 random position images were taken at 10, 20 or 40x magnification, and the total number of nuclei and their size were determined using the Stardist plugin for ImageJ (48). The obtained total number of nuclei was divided by the area of field of view to obtain the number of cells per cm^2^. Moreover, for each sample 3 random images were manually analyzed to determine the cell size, number of nuclei per cell and percentage of fusion (%Fusion; Equation 1).

**Table 2:** Antibodies used for fluorescent stainings.

Target	Primary	Conc.	Secondary	Conc.
F-actin	–	–	Phalloidin 550/647 (Sigma-Aldrich)	1-200
Tubulin	Rat anti-Human IgG2a (Novus Biologicals)	1-100	Goat anti-Rat IgG 488 (Invitrogen)	1-300
Vimentin	Mouse anti-Human IgM (Abcam)	1-100	Goat anti-Mouse IgM 647 (Jackson imm. Res.)	1-300

(1) $$\%Fusion=\frac{Number\;of\;nuclei\;within\;FBGCs}{Total\;number\;of\;nuclei}\times 100$$

Cell viability was confirmed by Calcein AM (Sigma-Aldrich)/Propidium Iodide (Biolegend) staining. Cells were washed with PBS and stained in MEMα medium without serum containing Calcein AM (1:1000) and Propidium Iodide (1:1000) for 5 minutes in an incubator. Subsequently, the well plate was placed in the Zeiss observer 7 (Carl-Zeiss) and a 3 by 4 millimeter area was imaged at 10x magnification to analyze viability.

### 2.5 Flow cytometry

A compensation matrix was created using anti-mouse compensation beads (BD Biosciences) and antibody-fluorophore conjugates with the same colors as detailed below. An aliquot of hPBMCs was taken after thawing to determine the monocyte purity. Polarized macrophages at day 0 were washed with PBS and detached using Accutase. hPBMCs or macrophages were washed in cold 1% BSA-PBS solution (PBA). Fc-receptors were blocked by incubating cells for 10 minutes in 2.5 µl trustain FcX (Biolegend) in 100 µL PBA at room temperature. Afterwards, cells were stained with CD80 (BV421; 1:50; Clone 2D10; Biolegend), CD206 (FITC; 1:25; Clone 15-2; Biolegend), IL-4Rα (APC; 1:25; Clone G077F6; Biolegend), IL-13R (PE/Cy7; 1:25; Clone SS12B; Biolegend), IL-2R/common γ chain (PE; 1:25; Clone TUGh4; Biolegend), CD11b (PerCP/Cy5.5; 1:25; Clone ICRF44; Biolegend), or CD3 (FITC; 1:25; Clone HIT3a; Biolegend) in PBA depending on the type of sample/control for 20 minutes on ice in the dark. Subsequently, cells were washed with PBA, resuspended in 400 µL PBA, measured on the flow cytometer (FACSCanto™ II; BD Biosciences), and analyzed using FlowJo. Fluorescence minus one (FMO) controls were used to determine expression of polarization marker and IL-4 receptor complex expression. To accomplish this, a positive gate for each marker was placed in the corresponding FMO control with a maximum of 1% positive cells (**Supplementary Figure 2**). This positive gate was used to determine the percentage of cells that expressed each epitope. Moreover, the geometric mean fluorescence intensity (GMFI) of the whole population of cells was measured to determine the amount of expression. To identify the purity of the monocyte ormacrophage populations, gates were set by identifying populations based on the SSC-A and FSC-a which was confirmed by CD11b and CD3 expression (**Supplementary Figure 3**).

### 2.6 ROS and oxidative stress quantification

The level of malondialdehyde (MDA; a by-product of oxidative stress) was measured using the lipid peroxidation assay kit (Sigma-Aldrich; MAK085) according to the supplier’s instructions. In short, cells were homogenized using 300 µL MDA lysis buffer and subsequently frozen at -80 °C until analysis. Once thawed, a reaction of MDA with thiobarbituric acid (TBA) was used to form a colorimetric product, which is proportional to the concentration of MDA present. To increase the sensitivity, 1-butanol was used to extract the TBA-MDA product. The absorbance was measured at 532 nm using a spectrophotometric multiwell plate reader (Synergy HTX multimode-reader; Biotek).

To semi-quantitatively measure reactive oxygen species, the 2’,7’-dichlorofluorescin diacetate (DCFDA) cellular ROS assay kit (Abcam; ab113851) was performed according to the supplier’s instructions. In short, cells cultured in a 96-well microplate (Cellvis; P96-0-N) were washed with 1x buffer and stained with DCFDA for 45 minutes in an incubator. Subsequently, DCFDA was removed and medium was added for 1 hour, except for the positive control which contained 150 µM tert-butyl hydroperoxide (TBHP) in 1x supplemented buffer. After 1 hour incubation, the fluorescence was measured at Ex/Em = 485/535 using a spectrophotometric multiwell plate reader (Synergy HTX multimode-reader; Biotek).

### 2.7 Gene expression profiles, RT-qPCR

Samples were washed with PBS, placed on ice, and subsequently lysed with 350 µL RLT-buffer in combination with scraping. For each biological replicate, two wells were pooled together to obtain sufficient RNA and further treated as 1 replicate, and the lysate was stored at -80 °C until further use. After thawing on ice, 350 µL 70% ethanol was added and mixed by pipetting. 700 µL sample was transferred to the RNeasy spin column and isolation was performed as described by the manufacturer (Qiagen). RNA concentration and purity was measured using a spectrophotometer (NanoDrop™ One/Onec Microvolume UV-Vis Spectrophotometer; ThermoFisher). cDNA was made in 20 µL reaction volume from 200 ng RNA by adding deoxynucleotide triphosphates (Invitrogen), random primers (Promega), 5x first strand buffer (Invitrogen), 0.1M dithiothreitol (Invitrogen), Moloney murine leukemia virus (M-MLV; Invitrogen), and RNA-free water. cDNA synthesis using a thermal cycle (C1000 touch, Bio-rad) was performed using the following heating steps: 65 °C for 5 min, on ice for 2 minutes (while adding the M-MLV mixture), 37 degrees °C for 2 min, 25 °C for 10 min, 37 °C for 50 min, 70 °C for 15 min, and 12 °C to cool down. cDNA samples were stored at -20 °C until further use for RT-qPCR. RT-qPCR was performed in a thermal cycler (CFX Opus 384; Bio-rad) using SYBR green supermix (Bio-rad) and water together with primers of genes involved in macrophage fusion, function, degradation, and tissue production (**Supplementary Table 1**). All samples were diluted 30-60x and pipetted with a technical replicate. The thermal protocol used consisted of: 95 °C for 3 min, a cycle of 95 °C for 20s, 60 °C for 20s, and 72 °C for 30 seconds repeated 40 times, 95 °C for 1 min, 65 °C for 1 min, and finally a melting curve measurement from 65 °C to 95 °C, incrementing 0.5 °C per cycle. Gene expression for the samples was normalized to the mean of the reference gene (β-actin) and a maximum allowable difference between values of technical replicates was set at 0.8 Ct.

### 2.8 Cytokine/Chemokine measurement; LEGENDplex™

The LEGENDplex™ Multi-analyte flow assay kit was used to quantify the amount of cytokines/chemokines produced. Two panels were purchased, the (1) human adipokine panel containing monocyte chemoattractant protein-1 (MCP-1), IL-1β, IL-10, IL-8, IL-6, IFN-γ, and tumor necrosis factor alpha (TNF-α); and the (2) human growth factor panel V02 containing transforming growth factor beta 1 (TGF-β1), platelet derived growth factor isoform BB (PDGF-BB), fibroblast growth factor 2 (FGF-2), and vascular endothelial growth factor (VEGF). The assay was performed according to the suppliers instructions. Important considerations were as followed: (1) for each biological replicate, the medium of 2 wells was pooled (i.e. 6 wells were combined into 3 replicates, i.e. n=3 per condition and per time point) and spun down at 14.000 rpm for 5 minutes. (2) Supernatant was then transferred and stored in -80 °C until used. (3) Lower reagent volumes in the same ratios were used. (4) The assay was performed the day before measurement, with the samples stored at 4 °C overnight. (5) Samples were measured using the FACSCanto™ II (BD Biosciences).

### 2.9 Statistical analysis

All data was statistically analyzed using GraphPad prism. Normality was tested using Shapiro-Wilk. For samples with multiple donors comparing two conditions, a paired t-test was performed. For comparing more than two conditions, repeated measures one-way ANOVA with Geisser-greenhouse correction and Tukey’s *post-hoc* was used, or Friedman test with Dunn’s *post-hoc* for samples that did not pass the normality check. Since the LEGENDplex™ data only contained one donor pool, samples were compared by ordinary one-way ANOVA with Tukey’s *post hoc*, or Kruskal-wallis test with Dunn’s multiple comparisons test for samples that did not pass the normality check. Lastly, for the MDA and DCFDA assay a two-way ANOVA with Šídák’s multiple comparisons test was used.

## 3 Results

### 3.1 Foreign body giant cells show distinct cytoskeletal organization

Macrophage polarization was confirmed using flow cytometry (**Supplementary Figure 1**) and the changes in cytoskeletal organization of differently polarized macrophages were investigated using immunofluorescent staining for F-actin, microtubules (tubulin), and intermediate filaments (vimentin) (**Figure 2A**). Specifically, pro-inflammatory M1 macrophages exhibited a more mesenchymal-like and elongated shape, whereas M2a and M2c macrophage retained a more rounded, amoeboid-like morphology. Additionally, the localization of actin-rich podosomes in M1 macrophages deviated from M2a and M2c macrophages, with many smaller, actin-rich patches present at the cell periphery in M1 macrophages (**Figure 2B**).

**Figure 2:**
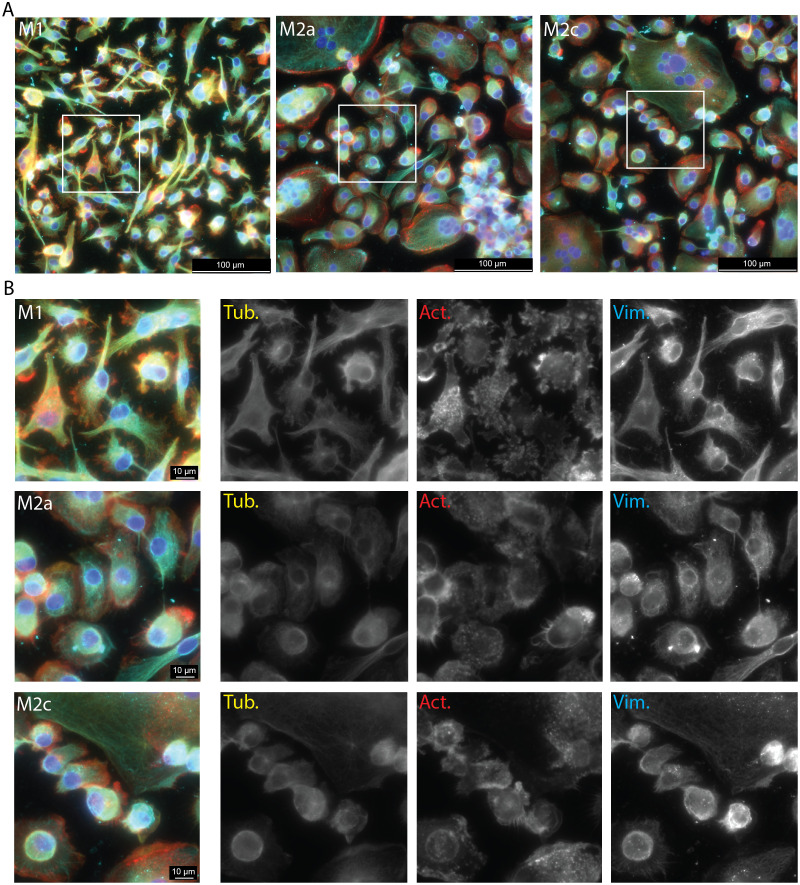
Cytoskeletal changes due to macrophage polarization. **(A)** representative images (donor D432) of polarized macrophages stained for tubulin (yellow), actin (red), vimentin (cyan), and nuclei (dark blue). Scale bars indicate 100 µm. **(B)** Zoomed-in areas indicated in **(A)** with fluorescent channels split for tubulin, actin, and vimentin. 40x magnification. Scale bars indicate 10 µm.

To investigate the changes in cytoskeleton from macrophages to FBGCs, fusion of unpolarized M0 macrophages was tracked over the first 14 days (Protocol A) (**Figure 3A**). Interestingly, spontaneous multinucleation (>2 nuclei) was already observed after 5 days of culture with M-CSF, prior to IL-4 stimulation (**Figure 3A**, Day 0). With the addition of IL-4, the number and size of FBGCs increased over time. Already after 2 days of IL-4 stimulation, the majority of macrophages appeared to have fused into multinucleated cells and only few singular macrophages were visible. Afterwards, FBGCs appeared to increase in size and number of nuclei. After 14 days of culture, a heterogeneous FBGC population was visible, varying in size and number of nuclei per FBGC. The largest FBGC reached a surface area of 220,000 µm^2^, as compared to a typical size range of 160-910 µm^2^ for singular macrophages.

**Figure 3:**
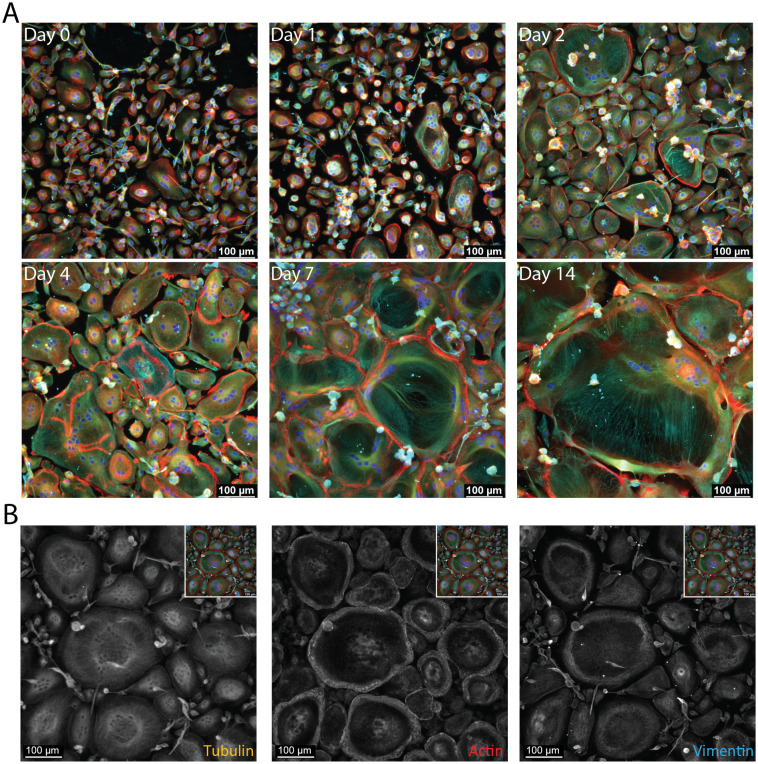
Cytoskeletal changes due to macrophage fusion. **(A)** representative images (donor D432) of macrophages and foreign body giant cells (FBGCs) from day 0 before interleukin-4 (IL-4) stimulation and after 1, 2, 4, 7, or 14 days of IL-4 stimulation. Cytoskeletal structures, tubulin (yellow), actin (red), and vimentin (cyan) and cell nuclei (dark blue) are visualized through fluorescent staining at 20x magnification. **(B)** FBGC structure visualized in more detail by splitting channels of tubulin, actin, and vimentin (D503). Scale bars indicate 100 µm.

Independent of their size, FBGCs showed a distinct cytoskeletal organization. Podosomes, which consist of an actin core, were found to be present at the edges of FBGCs, forming a ring. Additionally, an actin-rich area was present underneath the nuclei. Microtubules, containing tubulin, were arranged tightly surrounding the nuclei, with additional fibers directing toward the extremities of the FBGCs (**Figure 3B**). Similarly, intermediate filaments, stained by vimentin, were also oriented from the center of the cell toward the extremities, however, were more abundantly present at the edges of the FBGCs. Of the few mononuclear cells that were left, these seemed to mainly reside on top of the FBGCs, as a monolayer of FBGCs tended to develop. These mononucleated cells typically highly expressed both tubulin and vimentin.

### 3.2 Pilot data: spontaneous fusion is reduced in M1 versus M2a/M2c macrophages

The influence of polarization state on fusion was then investigated by polarizing macrophages for 2 days, followed by 7 days of IL-4-stimulated fusion (Protocol A) (**Figures 4A–D**). After polarization, the total number of nuclei was lower for M1/M2a/M2c polarization states (90-105*10^3^ nuclei/cm^2^) when compared to M0 macrophages (130*10^3^ nuclei/cm^2^) (**Figure 4B**; Day 0). 7 days of fusion resulted in a further decrease of the number of nuclei for all polarization states. Interestingly, M1 macrophage polarization resulted in a substantially larger decrease of nuclei, containing an average of 28*10^3^ nuclei/cm^2^ when compared to M2a and M2c macrophages (64 and 75*10^3^ nuclei/cm^2^, respectively).

**Figure 4:**
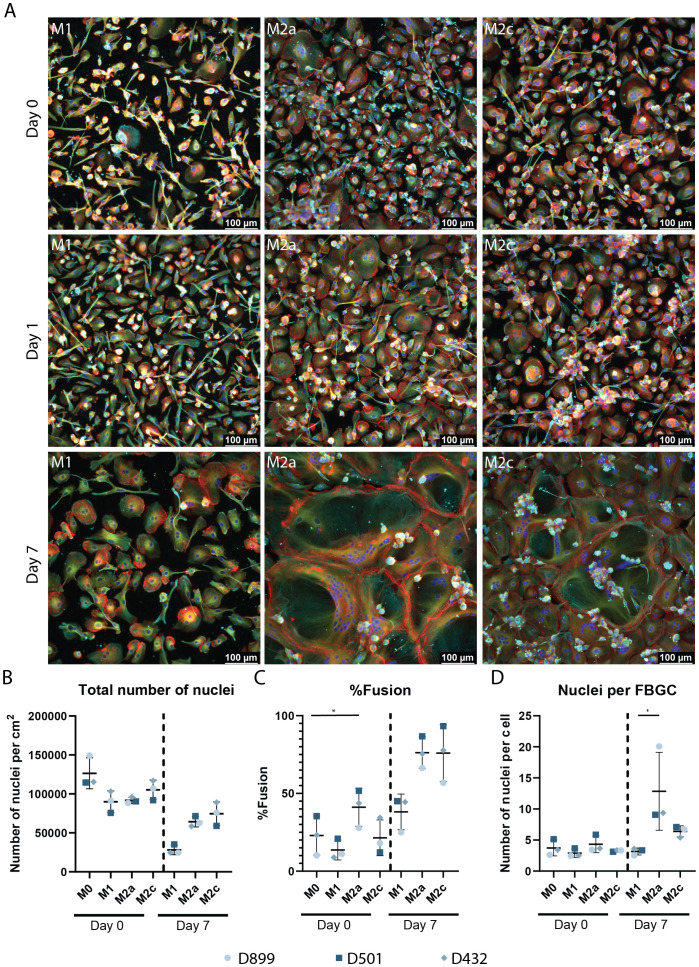
Effect of spontaneous fusion during macrophage polarization. **(A)** Macrophages stimulated toward the pro-inflammatory M1 phenotype, the anti-inflammatory M2a phenotype, and the regulatory M2c phenotype were imaged before interleukin-4 (IL-4) stimulation (Day 0) (donor D899 shown). Images display tubulin (yellow), actin (red), vimentin (cyan), and nuclei (dark blue). 20x magnification, scale bar indicates 100 µm. **(B)** Quantification of the total number of nuclei per cm^2^, **(C)** percentage of fused macrophages, and **(D)** nuclei per foreign body giant cell (FBGC) after polarization (Day 0) and after 7 days of IL-4 stimulation of pre-polarized macrophages. All metrics are quantified for 3 images of the same coverslip per donor, for 3 donors (n=3 biological replicates), with %Fusion calculated as number of nuclei within FBGCs/total number of nuclei *100%, and FBGCs identified as a cell with three or more nuclei.

Spontaneous fusion (13-40%) was noted before fusion induction (Day 0) (**Figure 4C**). This differed between polarization states, with M2a macrophages already showing an average of 40% fusion and M1 macrophages showing 13% fusion at day 0 (**Figure 4C**). After 7 days of IL-4 stimulation, all macrophage polarization states increased their fusion percentage further. M2a/M2c macrophages increased their fusion percentage to 75% compared to M1 polarized macrophages that reached a maximum average of 38%. A similar trend was observed for the number of nuclei per FBGC (**Figure 4D**). Especially after 7 days of fusion, FBGCs from M2a macrophages showed a significant increase in the number of nuclei per FBGC when compared to those from M1 macrophages.

Overall, these results revealed that M1 macrophages tend to show less fusion upon IL-4 stimulation when compared to M2a/M2c macrophages. However, these differences were already apparent before IL-4 stimulation, suggesting a strong confounding influence of spontaneous, contact-induced fusion on these findings. Furthermore, due to the addition of IL-4/IL-13 for M2a polarization, fusion was already substantially higher compared to the other polarization states. Aside from these effects of polarization on fusion, donor-to-donor differences were also noted, with some donors showing excessive fusion even before IL-4 stimulation (**Supplementary Figure 4**).

These important pilot data revealed a dominant influence of culture conditions on macrophage fusion, motivating the development of a modified culture protocol to enable a more direct comparison between polarization states at the moment of fusion induction via IL-4.

### 3.3 M1 macrophages leads to lowest percentage fusion, but largest FBGCs

In order to negate the effect of fusion prior to IL-4 stimulation, a modified protocol was developed that does not support spontaneous macrophage fusion but does support macrophage polarization (Protocol B; **Figure 1**). Macrophages from different donors were pooled to avoid donor-to-donor variability and fusion capabilities between donors was confirmed (**Supplementary Figure 5**).

Macrophage polarization at day 0 with protocol B was confirmed using flow cytometry (**Figure 5A**). CD80 (M1 marker) showed a mean percentage positive cells of 76% for M1 polarization, while M0/M2a/M2c macrophages showed 14-23% positive cells. Furthermore, the GMFI was around three times (2.9 vs 1.1) higher for M1 macrophages when compared to M2a/M2c polarized macrophages. The mean percentage of CD206 (M2a/M2c marker) positive M1 macrophages was 28%, while M0, M2a, and M2c macrophages showed 62%, 92%, and 73% CD206 positive cells, respectively. Moreover, M2a and M2c polarized macrophages showed a significantly increased GMFI of CD206 when compared to M1 polarized macrophages.

**Figure 5:**
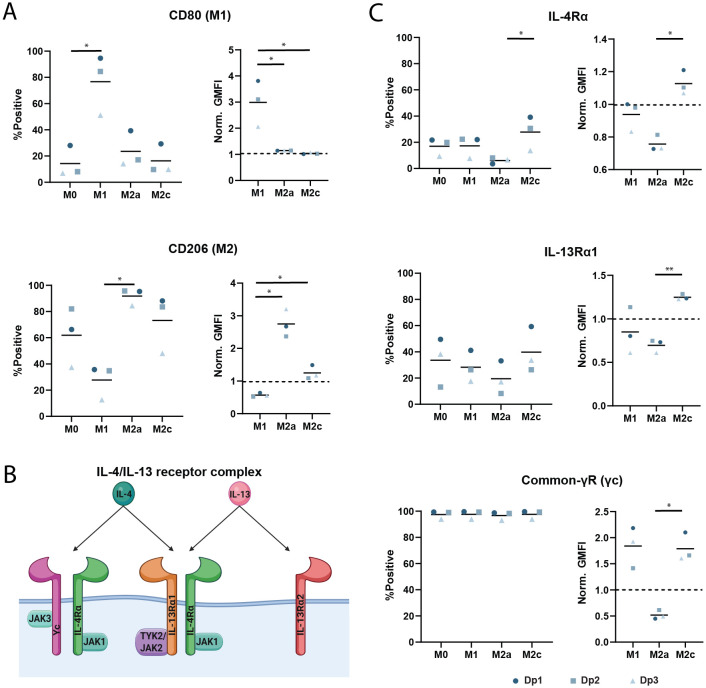
Macrophage polarization confirmation and interleukin-4 (IL-4)/IL-13 receptor complex expression measurement. **(A)** Polarization status of macrophages before initiating fusion (Day 0) was determined using cluster of differentiation 80 (CD80; M1) and CD206 (M2a). **(B)** Schematic of IL-4/IL-13 receptor complex; adapted from Nguyen et al., 2020 (53). Created in BioRender. Conner, T. (2026) https://BioRender.com/4h8j8pa. **(C)** Flow cytometry measurement of the three functionally active units of the IL-4/IL-13 receptor complex. Flow cytometry data shows the percentage of cells positive for the tested proteins and the geometric mean fluorescence intensity (GMFI) normalized to M0, for 3 donor pools (Dp1-3; n=3 biological replicates).

The expression of the IL-4/IL-13 receptor complex was measured for the various polarization states (**Figures 5B, C**). M2c macrophages showed a significant increase in both the percentage of positive cells and the GMFI when compared to M2a macrophages and showed approximately 10% higher expression when compared to M1 macrophages. The common gamma receptor showed a somewhat similar trend, with M2a macrophages showing a decreased expression when compared to M1 and M2c macrophages. However, close to all (97%) of the measured macrophages were positive for the common gamma receptor for all polarization states. IL-13Rα1 expression was highest for M2c polarized macrophages, with M1 and M2a macrophages showing similar expression. Similarly, the percentage of IL-13Rα1 positive cells was higher for M0 and M2c (34% and 40%, respectively) macrophages when compared to M2a and M1 macrophages (19% and 28%, respectively).

Genes that are linked to phenotype, ROS and enzyme production, cell fusion, and tissue production were analyzed using RT-qPCR (**Figures 6A–D**; **Supplementary Table 1**). Relative expression profiles showed a general trend of increased expression for M1 polarized macrophages across all M1-associated phenotypic markers (IL-6, chemokine (C-C motif) receptor 7 (CCR7), and TNF-α) (**Figure 6A**). Furthermore, these macrophages upregulated NADPH oxidase 2 (NOX2), nuclear factor kappa-light-chain-enhancer of activated B cells (NFκB), and TGF-β1 (**Figures 6B, D**). Interestingly, M1 macrophages also showed the highest relative expression for all used fusion-associated genes (CD44, signal-regulatory protein alpha (SIRPα), and purinergic receptor P2X7 (P2RX7)) (**Figure 6C**). M2a and M2c macrophages showed clear upregulations for CD206 and CD163, respectively (**Figure 6A**). Furthermore, lipase A (LIPA) expression was increased for M0, M2a, and M2c macrophages when compared to M1 (**Figure 6B**). MMP9 showed an increase in M2c macrophages when compared to M2a. Lastly, M2c macrophages also showed an increased tissue inhibitor of metalloproteinases 1 (TIMP1) expression when compared to all other macrophage polarization states (**Figure 6D**).

**Figure 6:**
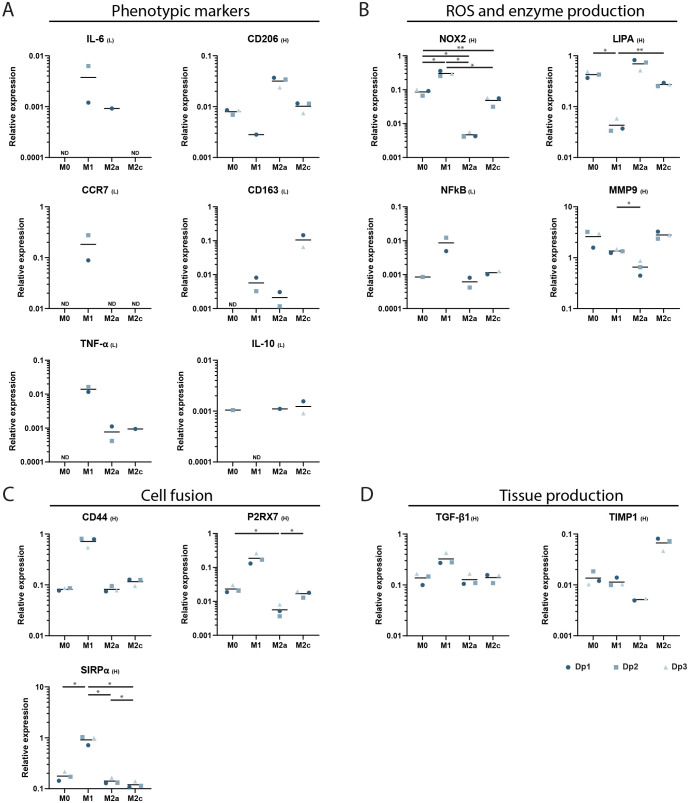
Effect of macrophage polarization on genes related to phenotypic markers **(A)**, reactive oxygen species (ROS) and enzyme production **(B)**, cell fusion **(C)**, and tissue production **(D)**. Gene expression relative to housekeeping genes of macrophages after 2 days of polarization. Each symbol represents a set of cells pooled from 6–9 donors (i.e. 6–9 donors treated as 1 biological replicate), with lines depicting the mean for 3 donor pools (Dp1-3; n=3 biological replicates). (_H_) and (_L_) depict dilution of cDNA by 60x or 30x, respectively. Samples depicted with ‘ND’ (not detectable) showed no signal or too high variability between technical replicates.

Aside from gene expression, protein expression was measured using a multiplex immunoassay (LEGENDplex™) (**Figure 7**). Note that IFN-γ and IL-10 were added to the media to induce polarization for M1 and M2c macrophages, respectively. M1 macrophages showed clear upregulation for MCP-1, IL-1b, IL-6, and TNF-α when compared to all other polarizations. Furthermore, although not as clear as the previously mentioned cytokines, IL-10 was higher in M1 macrophages compared to M0 and M2a macrophages. M2a and M2c macrophages showed a decreased trend of IL-8 expression when compared to M0 and M1 macrophages. Lastly, M2c macrophages showed significantly less IL-6 and TNF-α expression when compared to M2a macrophages. PDGF-BB, VEGF and FGF-2 were highly expressed in all conditions due to presence in the supplemented platelet lysate and were therefore excluded from further analysis.

**Figure 7:**
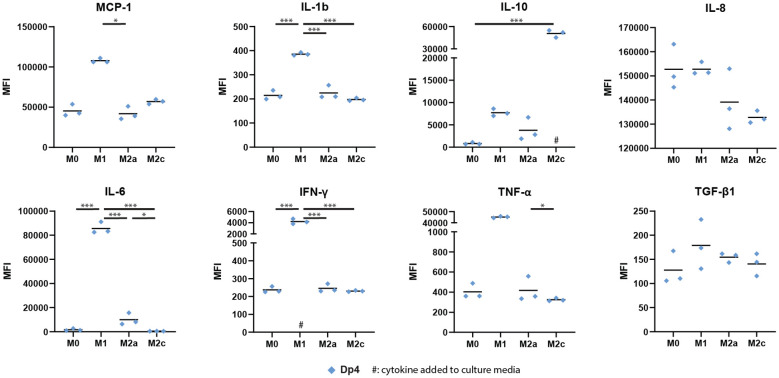
Protein expression of polarized macrophages. Cytokine/chemokine measurements using the LEGENDplex™ assay on medium from M0, M1, M2a, or M2c polarized macrophages. For each replicate, two wells were pooled into one sample, with three samples (i.e. n=3 replicates) for donor pool 4 (Dp4). Mean fluorescence intensity (MFI) shown with line as mean. # denotes samples where the specific cytokine was present in the polarization media. * p < 0.05, *** p < 0.001.

Cells were imaged after polarization (Day 0), fusion (Day 5), and re-polarization (Day 7) to examine the extent of fusion (**Figure 8A**). Again, pro-inflammatory M1 macrophages exhibited a more mesenchymal-like and elongated shape, whereas M2a and M2c macrophage retained a more rounded, amoeboid-like morphology before fusion. Similarly to protocol A, the total number of nuclei decreased during the culture; albeit starting at a lower number of nuclei per area (90-126*10^3^ nuclei/cm^2^ for protocol A vs. 64-96*10^3^ nuclei/cm^2^ for protocol B) for all polarization states except M1 macrophages, which was similar between protocols (90*10^3^ nuclei/cm^2^ for protocol A vs. 96*10^3^ nuclei/cm^2^ for protocol B) (**Figure 8B**). Specifically, the mean number of nuclei was highest for M1 macrophages at day 0 at 96*10^3^ nuclei/cm^2^ but decreased by 67% at day 7. In comparison, M0 macrophages started at 82*10^3^ nuclei/cm^2^ at day 0 and showed a 28% decrease. M2a macrophages had the lowest initial mean number of nuclei at 64*10^3^ nuclei/cm^2^, which decreased by 34% at day 7. Lastly, M2c macrophages showed the lowest decrease of 25% from the original 75*10^3^ nuclei/cm^2^ at day 0.

**Figure 8:**
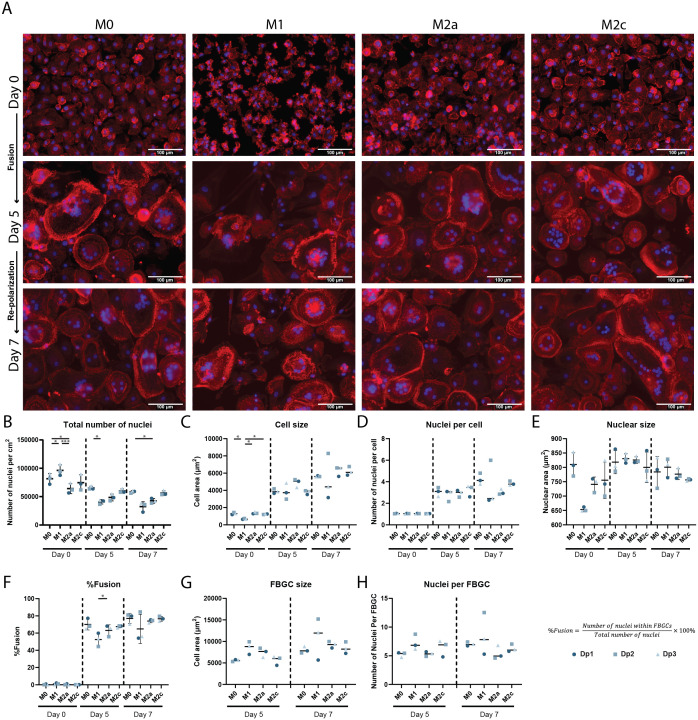
Foreign body giant cell (FBGC) formation quantification. **(A)** Non-polarized macrophages (M0) or stimulated toward the pro-inflammatory M1 phenotype, the anti-inflammatory M2a phenotype, and the regulatory M2c phenotype were imaged before interleukin-4 (IL-4) stimulation (Day 0), after IL-4 stimulation (Day 5), and after re-polarization (Day 7). Images stained for actin (red) and nuclei (dark blue). 10x magnification, scale bar indicates 100 µm. **(B)** Total number of nuclei per cm^2^ counted using the StarDist plugin for ImageJ on 10 images per sample shown as mean with SD. **(C, D)** Cell size and nuclei per cell measured manually using ImageJ on 3 parts of randomly selected images per sample, shown as median. **(E)** nuclear area counted using the StarDist plugin for ImageJ on 10 images per sample shown as mean with SD. **(F)** Percentage fusion (%Fusion), calculated using the equation shown in the bottom right of figure, with FBGCs classified as a cell with 3 or more nuclei, shown as mean with SD. **(G, H)** Size and number of nuclei per FBGC measured manually using ImageJ on 3 parts of randomly selected images per sample, shown as median. All data were measured for three different donor pools (Dp1-3; n=3 biological replicates). * p < 0.05.

All polarization groups showed similar trends for the size of the cells over time (**Figure 8C**). The mean area started at 678 µm^2^ for M1 macrophages and 1244-1373 µm^2^ for the others, and increased to an average of 4105 µm^2^ at day 5 and 5847 µm^2^ at day 7 for all polarization states. A similar trend was observed for the number of nuclei per cell (**Figure 8D**). Starting at a mean of 1 nucleus per cell at day 0, this increased to 3 nuclei at day 5 and 3–4 nuclei per cell at day 7 depending on the macrophage polarization state. Interestingly, nuclear size decreased in M1 macrophages and increased after fusion (**Figure 8E**). Histograms of cell size and number of nuclei per cell show a shift in the distribution of the population leading to a more heterogenous population when compared to day 0 (**Supplementary Figure 6**). Lastly, the percentage of fusion showed relatively similar trends for all polarization states from 0% at day 0 to 51-69% at day 5, with M1 macrophages showing a significantly lower amount of fusion when compared to M2a polarization states (**Figure 8F**). At day 7, fusion increased further to around 57-73% with M1 macrophages again showing a trend for lower fusion for two out of the three donor pools. Interestingly, the median FBGC size and number of nuclei per FBGC was highest for M1 FBGCs (**Figures 8G, H**).

### 3.4 Foreign body giant cells retain polarization-associated genes upon repolarization

To characterize the phenotype of FBGCs and re-polarized FBGCs, gene expression analysis was performed at day 5 and 7 (**Figure 9A**). Interestingly, after 5 days of fusion, all previously upregulated M1-associated genes were undetectable in the M1 FBGCs, suggesting a clear phenotypical switch as a result of fusion for M1 macrophages (**Figure 9A**). M2a/M2c-associated gene expression (CD206, CD163, IL-10), showed no clear trend for up or down regulation after 5 days of fusion between the different polarization states. After re-polarization (Day 7), M1-associated genes again showed upregulation in M1 FBGCs. After re-stimulation with both M2-associated stimuli, the changes were not as profound as in initial polarization (**Figure 6A**), although IL-10 expression tended to be upregulated for both M2-type FBGCs when compared to M0 and M1.

**Figure 9:**
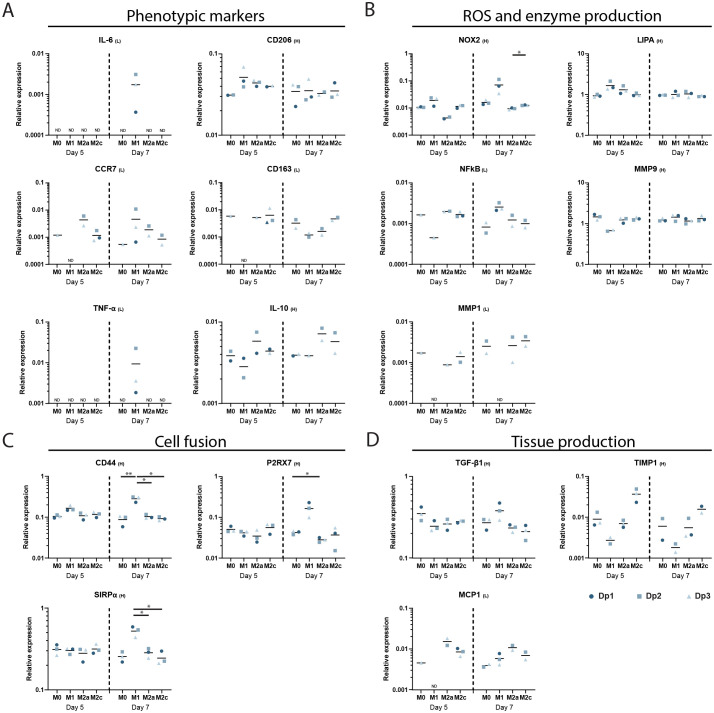
Effect of macrophage polarization on foreign body giant cell (FBGC) polarization. Genes related to phenotype **(A)**, reactive oxygen species (ROS) and enzyme production **(B)**, cell fusion **(C)**, and tissue production **(D)** were measured. Gene expression relative to housekeeping genes. (_H_) and (_L_) depict dilution of cDNA by 60x or 30x respectively. Samples depicted with ND showed no signal or too high variability between technical replicates. Day 5 represents five days of interleukin-4 (IL-4) induced fusion after polarization and day 7 represents two days of re-polarization of FBGCs. All lines in the figures show mean value with each shape representing a different donor pool (Dp1-3; n=3 biological replicates). * p < 0.05.

Aside from these typical phenotypical macrophage markers, genes related to ROS and enzyme production were measured to reflect the degradative capacity of FBGCs (**Figure 9B**). Gene expression after fusion showed a trend for increased NOX2 and LIPA expression in M1 FBGCs when compared to the other polarization states. Furthermore, a trend for a decreased NFκB and MMP9 expression was seen for M1 macrophages when compared to all other polarization states.

When compared to M1 macrophages at day 0, fusion related genes showed no upregulation in M1 FBGCs anymore after fusion (Day 5), except for a small trend in CD44 (**Figures 6C**, **9C**). However, when re-stimulated, similar expression patterns to unfused M1 macrophages were detected, with significant increases in M1 FBGCs when compared to the other polarization genes.

Lastly, genes related to tissue production were measured (**Figure 9D**). When compared to macrophages at day 0 (**Figure 6D**), TIMP1 at day 5 again showed increased expression for M2c FBGCs when compared to M0 and M2a polarization, with an even stronger decrease for M1 FBGCs. The previously shown upregulation of TGF-β1 in M1 macrophages (**Figure 6D**) was lost after fusion (**Figure 9D**). However, after re-stimulation, TGF-β1 reaquired an increase for M1 FBGCs. Furthermore, after re-stimulation, MCP1 remained to show trends for increases in gene expression for M2a FBGCs.

To confirm RT-qPCR findings, protein and ROS measurements were performed as readouts of key FBGC functions (**Figure 10**). Note that the increased values for IL-10 and IFN-γ for M2c and M1 re-polarization (Day 7), respectively, are due to the addition of these factors in the polarization cocktail (**Figure 10A**). Similar to the RT-qPCR data, IL-6, TNF-α, and TGF-β1 secretion were higher in M1 stimulated FBGCs (Day 7) when compared to other polarization states. Furthermore, M1-stimulated FBGCs significantly upregulated MCP-1 when compared to the other polarization states. Moreover, FBGCs from M1 polarized macrophages (Day 5) showed a significant decrease in IL-10 expression compared to all other polarization states, which carried over after re-polarization. FBGCs from M2c polarized macrophages (Day 5) showed an increased IL-8 expression when compared to all other polarization states. However, after re-polarization (Day 7) M1 polarized FBGCs overtook the IL-8 expression when compared to all other polarization states, with M2c still showing an increase over M0 and M2a polarized macrophages. ROS expression was determined using two assays that measure ROS in different ways (**Figure 10B**). The assay results showed a clear trend of increased ROS expression after re-polarization (Day 7) when compared to day 5 for all polarization types. Similarly, both assays showed that FBGCs from M1 polarized macrophages resulted in the lowest amount of ROS expression.

**Figure 10:**
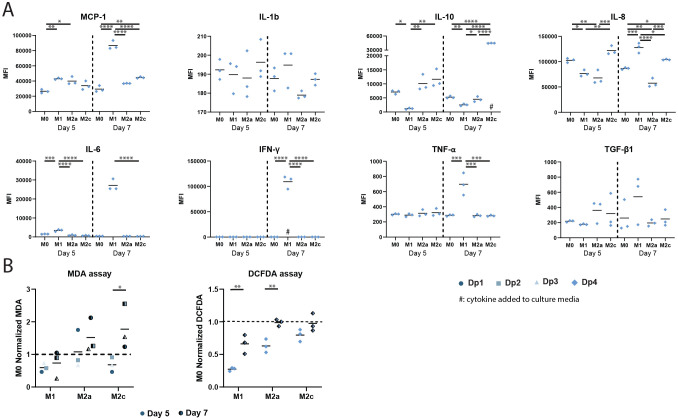
Protein expression and reactive oxygen species (ROS) measurements of foreign body giant cells (FBGCs). **(A)** Cytokine/chemokine measurements using the LEGENDplex™ assay on medium from FBGCs that were fused from polarized macrophages (Day 5), and after re-polarization (Day 7). Two wells were pooled into one sample, leading to n=3 replicates, all from donor pool 4 (Dp4). Mean fluorescence intensity (MFI) shown with line as mean. # denotes samples where the specific cytokine was present in the polarization media. **(B)** visualization of the concentration of malondialdehyde (MDA; byproduct of oxidative stress) and 2’,7’-dichlorofluorescin diacetate (DCFDA; Dye that becomes fluorescent under oxidation by ROS) normalized to M0 on day 5 and day 7. MDA performed on Dp1–3 and DCFDA assay on Dp4, with lines depicting mean in both graphs. * p < 0.05, ** p < 0.01, *** p < 0.001, **** p < 0.0001.

## 4 Discussion

The goal of this study was to investigate the effect of polarization state on fusion of primary human macrophages, and whether fused macrophages retain phenotypical features of their previous polarization state. We demonstrate that FBGCs can form from all macrophage polarization states, although M1 polarization leads to a decreased fusion percentage. However, the fewer FBGCs that form from M1 macrophages display larger FBGCs with more nuclei per cell. Phenotypically, FBGCs reacquire macrophage polarization-state associated gene expression and cytokine secretion upon restimulation with polarization-inducing stimuli, in particular for M1-associated polarization. These findings suggest that FBGCs are able to change their phenotype depending on their environment, similar to mononuclear macrophages, and that their function can be steered.

The results presented here oppose our initial hypothesis that IL-4-induced FBGC formation would be mainly driven from M1 macrophages. Flow cytometry analysis showed that M1 macrophages indeed express higher levels of the receptors for IL-4 when compared to M2a macrophages, corroborating the findings of O’Brien et al. and Fenn et al. (52, 54). Interestingly, our results showed that M2c macrophages have an even higher expression of IL-4 receptors. In addition to increased IL-4 receptor levels, M1 macrophages showed an upregulation of CD44, P2RX7, and SIRPα, which have been proposed to be essential for fusion (55–57). Surprisingly, the increased expression of IL-4 receptors and fusion-related genes in M1 macrophages did not lead to increased levels of IL-4 induced fusion, and, in fact, to decreased fusion. This paradoxical finding may be explained by the notion that the transmembrane presence of IL-4R is dependent on IL-4 concentration and time. Research in T- and B-cells has shown increased IL-4R expression after IL-4 addition and a study that performed western blotting on IL-4 stimulated macrophages reported increased IL-4Rα and IL-13Rα1 protein expression (58). A decrease of IL-4R over time in conditions with high levels of IL-4 was also suggested to be caused by internalized IL-4/IL-4R complexes (59, 60). As all flow cytometry measurements in the present study were performed on non-permeabilized cells, it is possible that binding of IL-4/IL-13 to the receptor complexes resulted in internalization of this receptor, causing the lower measured expression of the IL-4/IL-13 receptor complexes in M2a macrophages.

Aside from the receptor complexes that are required for IL-4 induced fusion, gene expression of cell fusion related markers were also measured. However, the here tested fusion-associated genes have also been linked to other immunological functions. For example, CD44 upregulation in response to LPS has been postulated to be important for migration of monocytic cells to sites of inflammation (61). Likewise, P2RX7 has been linked to inflammasome activation, membrane permeabilization, and uptake of large molecules (62). On the other hand, SIRPα has also been suggested to play a role in preventing hyperactivation under proinflammatory conditions (63). Van Putten et al. previously reported that contamination of collagen scaffolds with LPS delayed the FBR and macrophage fusion after implantation in rats (64), which is in line with our *in vitro* findings. They proposed subsequent upregulation of IL-10 after bacterial endotoxins might have caused delayed fusion. Yet, our findings showed that direct IL-10 stimulated M2c macrophages did not exhibit delayed or inhibited fusion.

The observed reduction in fusion of M1 macrophages may be a direct effect of metabolic programming during polarization, as intrinsic differences are known between pro- and anti-inflammatory macrophages in terms of metabolism. M1 macrophages have been reported to rely mainly on inefficient glycolysis for rapid ATP supply as this also provides metabolic intermediates which are crucial for cell adaptation to sustain pro-inflammatory mediator production (65–67). In contrast, M2 macrophages rely mainly on oxidative phosphorylation for sustained energy (66, 68). Specifically, previous research has shown that LIPA, which is essential for intracellular lipid metabolism in macrophages, is upregulated in IL-4 polarized macrophages and fueling the fatty acid oxidation (68–70). Previous work has shown that hyperglycemia influences the response of human macrophages to biomaterials *in vitro* (71). In the context of fusion, Lemma et al. suggested that fusion in osteoclasts mainly relied on oxidative phosphorylation whereas bone resorption relied on glycolysis (72). This may indicate that M1 macrophages relied too much on the glycolysis pathway and did not have the ideal energy source available for fusion. The increased ROS-associated NOX2 expression at day 0 for M1 polarized macrophages indeed hints at the fact that these macrophages were actively engaged in glycolysis-mediated tasks (73). This is further supported by the decreased LIPA expression in M1 macrophages compared to all other macrophages phenotypes. However, after fusion, M1 pre-polarized FBGCs appeared to show an increased LIPA expression, indicating a possible change in metabolic activity. Furthermore, providing FBGCs with M1 stimulating factors did not reduce LIPA activity, possibly due to the different metabolic requirements for fusion. Further studies into the metabolic needs of macrophages during fusion and functioning of FBGCs would be needed to verify these hypotheses.

Apart from differences in metabolism, macrophage polarization with biochemical cues also leads to cytoskeletal changes, as also observed in this study. For example, actin polymerization has been reported to be required for macrophages in order to produce pro-inflammatory cytokines in response to LPS (74). Macrophage fusion involves extensive cytoskeletal reorganization and the migration of podosomes into ‘zipper-like’ structures at the cell-cell interface (32, 33). For osteoclasts it has been reported that differences in cytoskeletal organization of integrins and actin, as a result of the local cellular environment, affects fusion (75, 76). Hence, the differences in cytoskeletal organization between the various macrophages types may contribute to the differences in fusion as observed.

In addition to intrinsic differences in fusion potential between macrophage polarization state, it is likely that the reduced cell numbers in the M1 macrophages affected the percentage of fusion. The secreted cytokines and oxidative stress (as reflected for example in the increased NOX2 expression) of M1 polarized macrophages may have compromised cell viability before fusion, also explaining the significant decrease in cell numbers from day 0 to 5 in this group. However, live/dead staining with calcein and propidium iodide did not show substantial cell death in any of the conditions (**Supplementary Figure 7**). This could also explain why larger FBGCs are formed from the M1 polarized macrophages; perhaps the lower density of macrophages resulted in less confinement, leading to larger FBGCs with more nuclei. Furthermore, these differences in cell density should also be noted in regards to the ROS measurements (i.e. MDA and DCFDA assays), as both of these assays follow the same trajectory as the number of nuclei.

Formation of multinucleated giant cells by macrophages is a complex, not well understood, multistep process including acquirement of a fusion competent state, chemotaxis and cell-cell adhesion, and finally fusion (30). Research by Ruder et al, has shown that something as simple as the culture density significantly influences the functional phenotype of macrophages (77). Moreover, it has been suggested that fusion-competency can be acquired by cell-cell contact, independent of IL-4/IL-13 stimulation (30, 78, 79). Hence, a lower cell density may indirectly affect fusion percentage. This is supported by our finding that macrophages showed rather extensive spontaneous fusion in MEMα media even without exogenous IL-4 stimulation, which we speculate to be caused by cell-cell contact-initiated fusion competency. This also implies that the fusion as we measured in this study is most likely to be the combined result of DAP12 signaling, initiated by cell-cell contact, and IL-4-dependent fusion via IL4 receptor activation (30, 31).

To avoid excessive fusion prior to IL-4 stimulation, a modified fusion protocol was used that utilized the fusion inhibition of RPMI-1640 during monocyte-to-macrophage differentiation and macrophage polarization. This two-step protocol enabled the use of equal conditions between experimental groups in terms of fusion percentage at the moment of IL-4 stimulation to induce fusion (Day 0). For future studies it may be beneficial to also seed a similar amount of polarized macrophages in order to negate the effects of differing cell numbers. The finding that RPMI-1640 media did not accommodate fusion was surprising as RPMI-1640 is one of the most commonly used media to culture FBGCs *in vitro* (80). In our hands, RPMI did not allow for relevant levels of macrophage fusion from primary human macrophages, even under increasing cell seeding densities, increasing IL-4 concentrations or changing culture surfaces (data not shown). This emphasizes previous calls for standardization of FBGC culture protocols *in vitro* (80, 81). Further investigations are needed to determine what specific components of RPMI-1640 and MEMα are important for the change in cellular conditions which either inhibits or supports fusion.

Apart from the finding that macrophage polarization state influenced fusion, an important finding of this study was that FBGCs are able to reacquire polarization state-associated gene and protein expression profiles upon restimulation with biochemical polarization cues. This was most profound for M1-derived FBGCs, which reacquired M1-associated gene and cytokine expression (TNF-α, CCR7, IL-6, MCP-1) upon restimulation with LPS/IFN-γ. An *in vivo* study by Zhen et al., which examined synthetic vascular grafts implanted in sheep, showed that FBGCs could express M1- and/or M2-type markers (82). Similarly, in a previous *in vivo* study by our own group using resorbable synthetic heart valves in sheep, FBGCs were detected with varying expression levels of arginase 1 and inducible NOS (iNOS), indicating a heterogeneous population (34). These results all point to the possibility that FBGCs may be able to polarize similarly to macrophages. Interestingly, the increased NOX2 and NFκB gene expression in M1 FBGCs upon re-stimulation was accompanied by an increased TGFβ1 gene and cytokine expression, which may suggest that TGFβ1 synthesis is dependent on ROS, as previously suggested (83). To determine ROS production, MDA and DCFDA levels, as indirect measures for ROS production, were measured and showed a clear trend of being upregulated in FBGCs upon re-stimulation for all macrophage polarizations. This was accompanied by a clear trend for upregulating TIMP1, which is linked to inhibition of MMPs (84). Interestingly, the expression of TIMP1 was sustained during fusion and remained upregulated upon restimulation. Similarly IL-10 and IL-8 secretion were significantly different dependent on the pre-polarization. These findings indicate that FBGCs may also partly preserve their previous phenotype. Taken together, the observed phenotypical plasticity of FBGCs, stemming from differently polarized macrophages and in different biochemical conditions, warrants further investigation into how these phenotypical variety translates to differences in FBGC function, particularly for key FBGC functions such as biomaterial resorption and coordination of fibrous encapsulation. Experiments have been performed to try and quantify the amount of degradation of biomaterials by FBGCs using scanning electron microscopy (**Supplementary Figure 8**) or weighing (data not shown). However, as the whole process of macrophage fusion was also performed on the biomaterial, degradation was found in every condition, even before fusion. Furthermore, no adequate way of quantifying the results has been found so far. In regards to fibrous encapsulation, expression of pro- or anti-fibrotic factors by FBGCs, such as IL-10 and TGF-β1, indicate that FBGCs may directly influence fibroblast activation, as has been indicated previously (85).

Several limitations should be taken into account when interpreting the results presented here. In terms of macrophage polarization, it is well acknowledged that the widely used *in vitro* M1/M2a/M2c paradigm, as also employed here for pragmatic reasons, is oversimplified and does not fully recapitulate the variety of macrophage phenotypes *in vivo*. Particularly, the use of LPS as M1 stimulus is of limited relevance when considering the case of emulating *in vivo* sterile inflammation like found during biomaterial implantation. However, this approach was chosen because LPS stimulation is by far the most well-characterized macrophage phenotype *in vitro* and *in vivo*. An interesting alternative would be to stimulate macrophages with damage associated molecular patterns, which have been postulated to be released upon implantation of biomaterials and possibly interact with the biomaterial, thereby activating macrophages into their M1 phenotype (86). To add to this, all cultures were performed on tissue culture plastic and glass coverslips. Based on previous reports, it is possible that the formation and phenotype of FBGCs is dependent on the substrate (87). Therefore, investigating the findings presented here on different substrates could indicate whether these findings are generic across various materials, or material-specific. With regards to inflammatory settings, the pooling of macrophages derived from hPBMCs that still contain some other immune cells, may result in an immune response. The protocol employed here, resulted in a macrophage purity of 83-91%, for three tested donors (**Supplementary Figure 3**). This immune response may activate inflammatory pathways in the macrophages, however the genes/cytokines tested here only show an upregulation when provided with LPS/IFN-γ. Investigating the exact types of cells present and their activation state may help deciphering whether this response significantly alters macrophage activation states. As FBGC formation was found in both single donor experiments, and experiments with pooled donors, this does not appear to significantly influence FBGC formation. Lastly, a demographic skew in the donor cohorts is present, with no sex- or age-disaggregated analysis performed. Previous reports suggest that patient characteristics may influence the response to implanted biomaterials, such as vascular grafts, although data is sparse (88–90). Future studies would be needed to systematically investigate the effects of these factors on FBGC formation. However, from our observations during FBGC cultures, it appeared that the limited data that blood suppliers are allowed to provide (i.e. sex, age, and blood type) does not appear to significantly alter FBGC formation. It is likely, that other factors such as smoking, weight, immune activation, or other non-severe comorbidities that still allow blood donation have a larger influence on FBGC formation.

## 5 Conclusion

This study shows that FBGCs can arise from different macrophage polarization states, with M1 polarization leading to fewer but larger FBGCs. Moreover, several phenotypical traits that are associated with specific polarization states are either preserved during fusion, or reacquired in polarization state-associated biochemical environment, providing evidence that FBGCs show at least some form of memory of pre-polarization, as well as the possibility to be induced into different polarized states as those found in macrophages. This implies that FBGC function may be steerable, similar to mononuclear macrophages, for example using biomaterial design parameters. Subsequent *in vitro* models closely mimicking the physiological microenvironment of implanted biomaterials would be a valuable next step to further investigate how biomaterial design characteristics influence FBGC formation and functionality.

## Data Availability

The datasets presented in this study can be found in an online repository. The name of the repository and accession number can be found below: Macrophage Polarization State Influences Fusion and Phenotype of Human Foreign Body Giant Cells In Vitro, 4TU.ResearchData, https://doi.org/10.4121/deab2be6-b27d-49e3-a442-cc3f1eb4d5dd.
