# Supplementary material for: Macrophage polarization state influences fusion and phenotype of human foreign body giant cells in vitro
Source: Front Immunol. 2026 Jul 21;17:1832686. doi: 10.3389/fimmu.2026.1832686 (PMC13433290; doi:10.3389/fimmu.2026.1832686)
Supplement: Supplementary file 1 [file DataSheet1.pdf]

Supplementary Material

Table S1. Genes analyzed via RT-qPCR. Adapted from Wissing et al., 2019. DOI: 10.3389/fbioe.2019.00087

Gene	Symbol	Function
**Phenotypic markers**		
Interleukin 6	IL-6	Pro-inflammatory factor
Chemokine (C-C motif) receptor 7	CCR7	Pro-inflammatory macrophage marker
Tumor necrosis factor alpha	TNF-α	Pro-inflammatory factor, stimulus for collagen production, inhibitor of elastogenesis
Mannose receptor c, type 1	CD206	Anti-inflammatory macrophage marker
Cluster of differentiation 163	CD163	Anti-inflammatory macrophage marker
Interleukin 10	IL-10	Anti-inflammatory cytokine, inhibitor of collagen production
**Cell fusion**		
Cluster of differentiation 44	CD44	Cell adhesion, migration, and fusion
Signal regulatory protein alpha	SIRPα	Cell fusion and phagocytosis
Purinergic receptor P2X7	P2RX7	Regulator of caspase activity, cytokine secretion, cell apoptosis and fusion
**ROS and enzyme production**		
Nicotinamide adenine dinucleotide phosphate oxidase 2	NOX2	Enzyme complex that contributes to ROS production
Lipase A or cholesterol ester hydrolase	LIPA	Lysosomal enzyme
Nuclear factor kappa-light-chain-enhancer of activated B cells	NFκB	Involved in cellular responses to oxidative stress and survival
Matrix metalloproteinase 9	MMP-9	Anti-inflammatory factor involved in extra-cellular breakdown and remodeling
Matrix metalloproteinase 1	MMP-1	Extra-cellular breakdown and remodeling, colla genase
**Tissue production**		
Transforming growth factor beta 1	TGF-β1	Anti-inflammatory factor; stimulus for collagen formation
Metallopeptidase inhibitor 1	TIMP-1	Inhibitor of MMPs
Monocyte chemoattractant protein 1	MCP-1	Chemotactic for monocytes/macrophages

Supplementary Figures

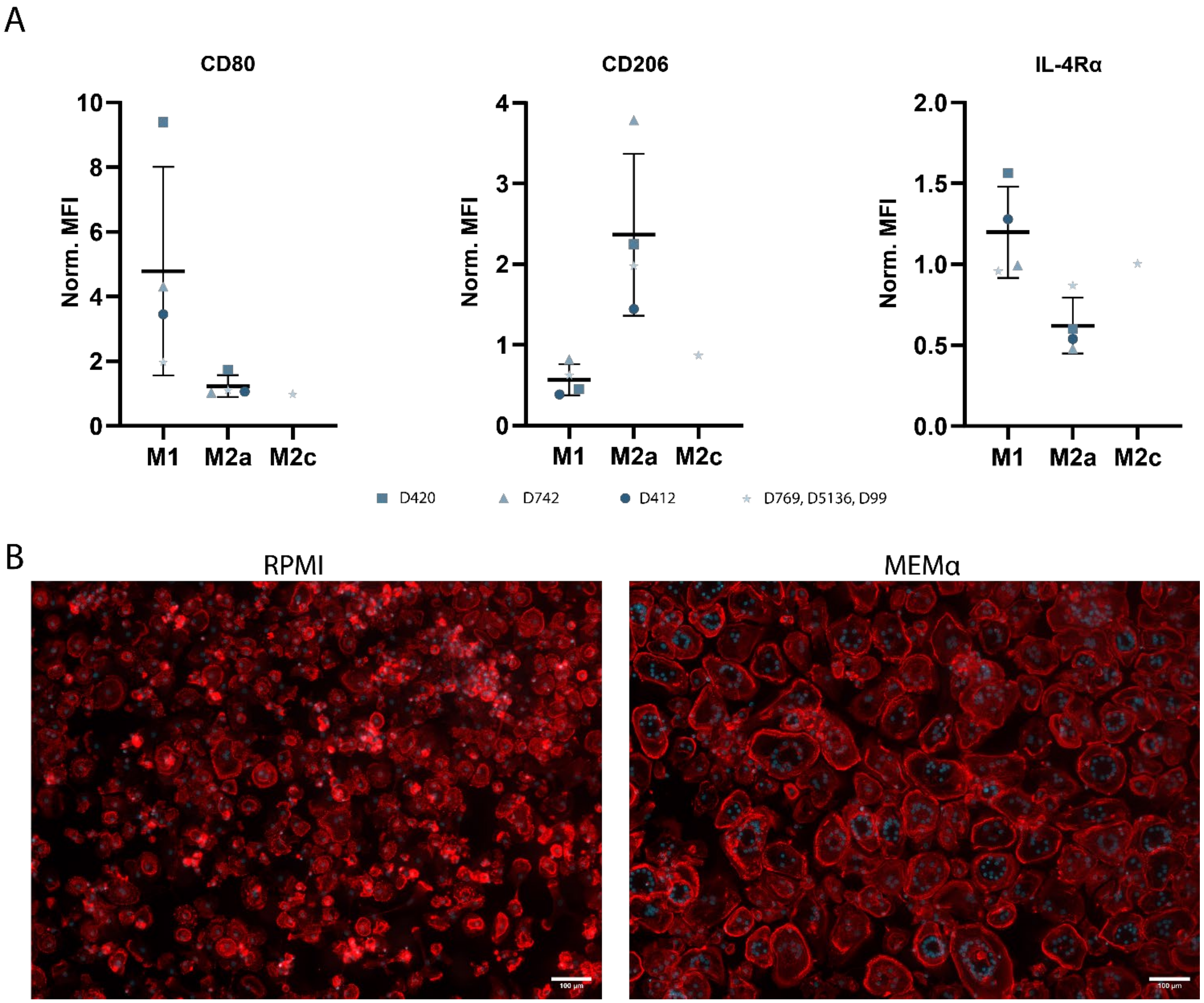

Figure S1. Effects of culture medium on polarization and fusion. (**A**) Flow cytometry data from cells cultured in MEMα medium for the first 7 days. All data normalized to M0 macrophages from the same donor. Donor represented by a star is data from 3 donors that were cultured separately and were measured as a pool during flow cytometry. Graphs show means ± SD. (**B**) Example image from the difference in fusion between macrophages cultured in complete RPMI + interleukin-4 (IL-4) or complete MEMα + IL-4 for 5 days. Nuclei (DAPI) stained in blue, and f-actin (phalloidin) stained in red. Scale bars represent 100 μm.

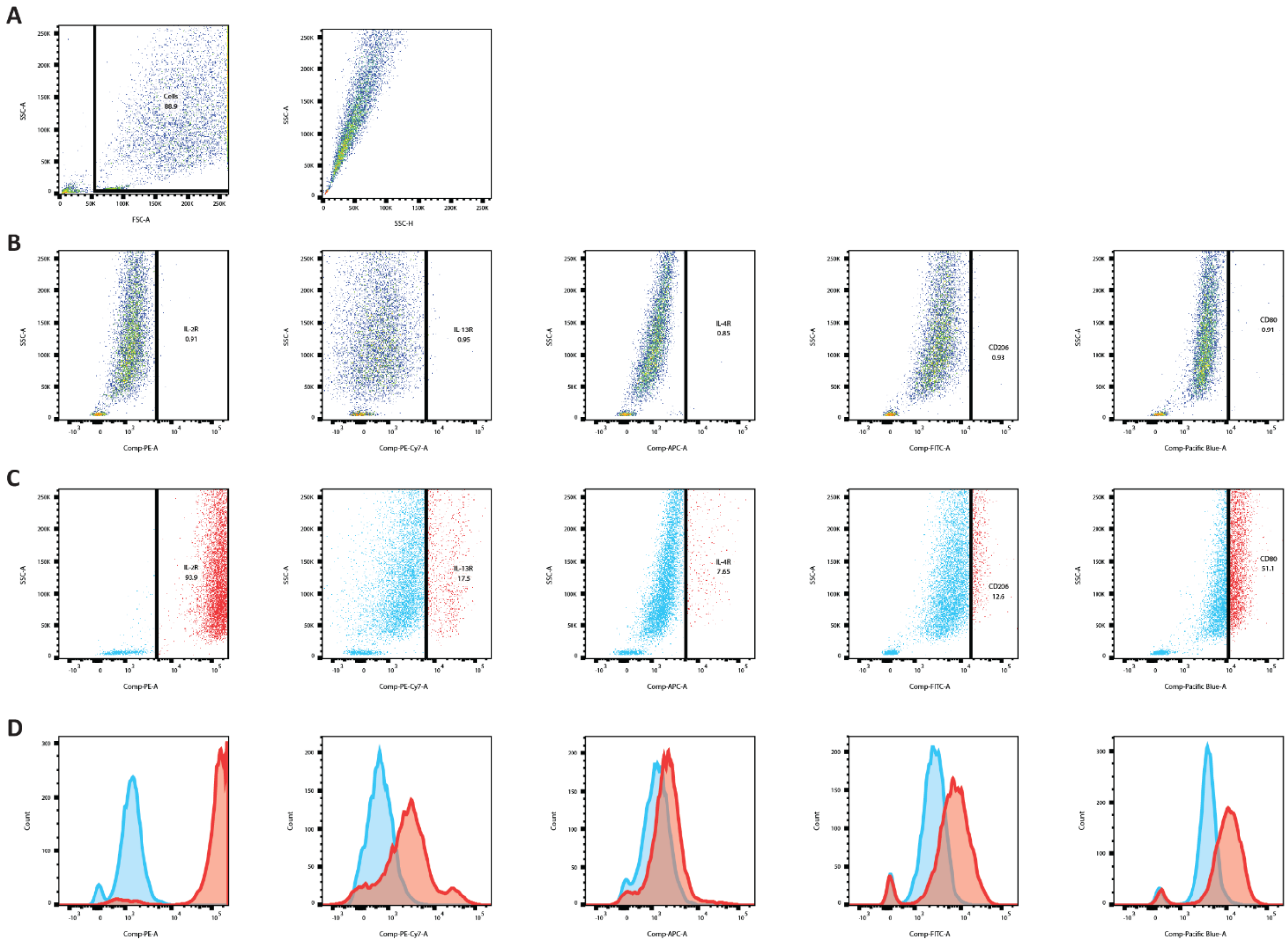

Figure S2. Gating strategy for figure 5. (**A**) Cells were gates from the SSC-A and FSC-A, with no gating for single cells. (**B**) Gates were placed in the corresponding fluorescence minus one (FMO) controls with a maximum of 1%. (**C**) Example of M1 FMOs being used to measure the number of positive cells. Blue dots represent the cells that are negative for the marker, and red dots represent positive cells. (**D**) Example of histograms that were used to measure the geometric mean fluorescence intensity. Blue represents the unstained control, red represents the stained sample (M1 in this example).

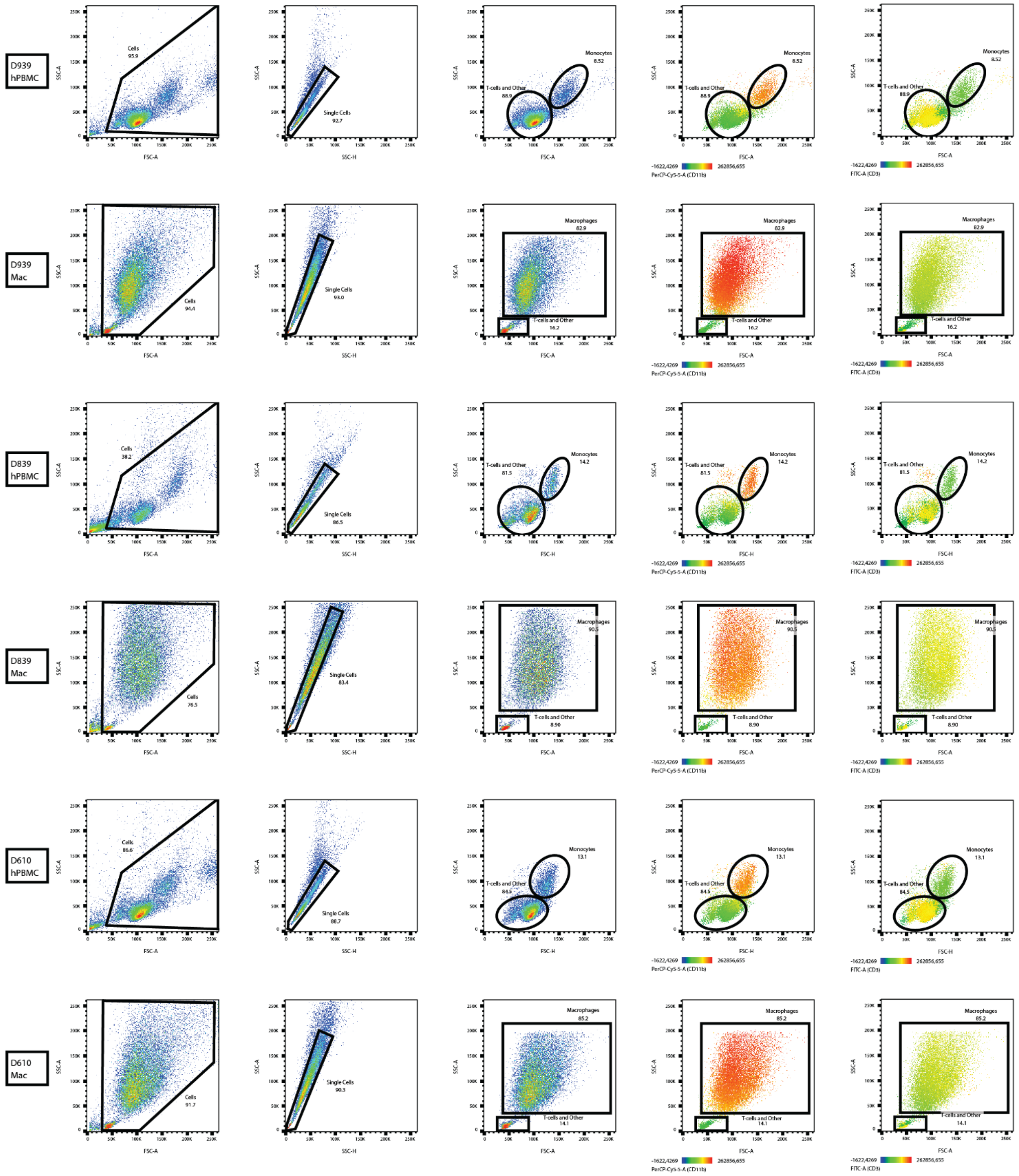

Figure S3. Gating strategy to determine monocyte/macrophage purity before and after differentiation. Purity of monocytes/macrophages was tested before seeding (hPBMC) and after detaching (Mac) using CD11b and CD3. Each row contains the same set of graphs from left to right: gating for cells, gating for single cells, gating for monocytes/macrophages and other cell types, CD11b signal (red = high, green = low), CD3b signal (red = high, green = low). Three donors were tested, denoted by a "D" followed by three numbers.

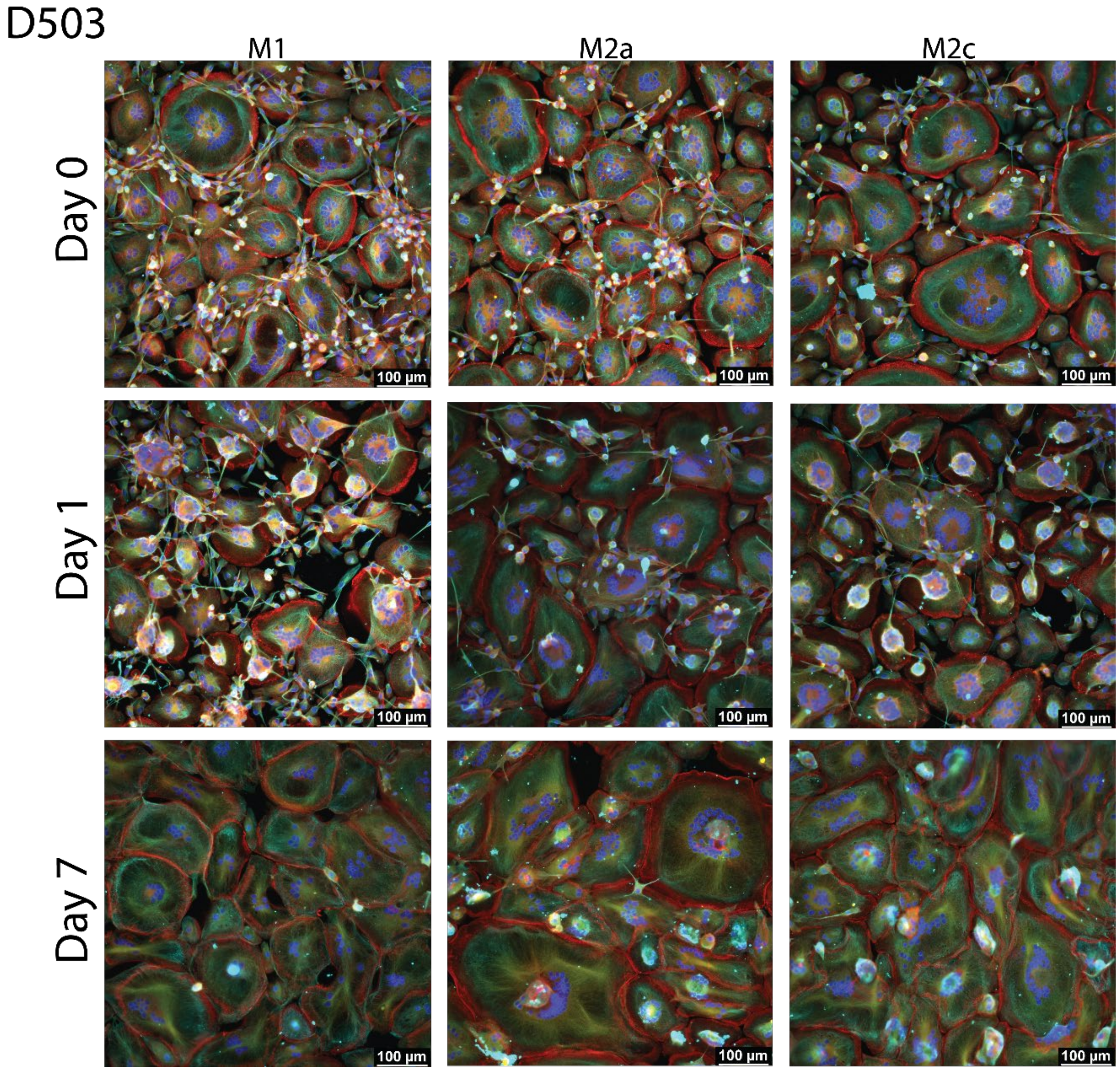

Figure S4. Excessive fusion of donor 503 before interleukin-4 (IL-4) stimulation. For all polarization conditions and timepoints (before IL-4 stimulation and after IL-4 stimulation) macrophage multinucleation was present. Staining for actin (red), tubulin (yellow), vimentin (cyan), and nuclei (dark blue). Scale bars indicate 100 µm.

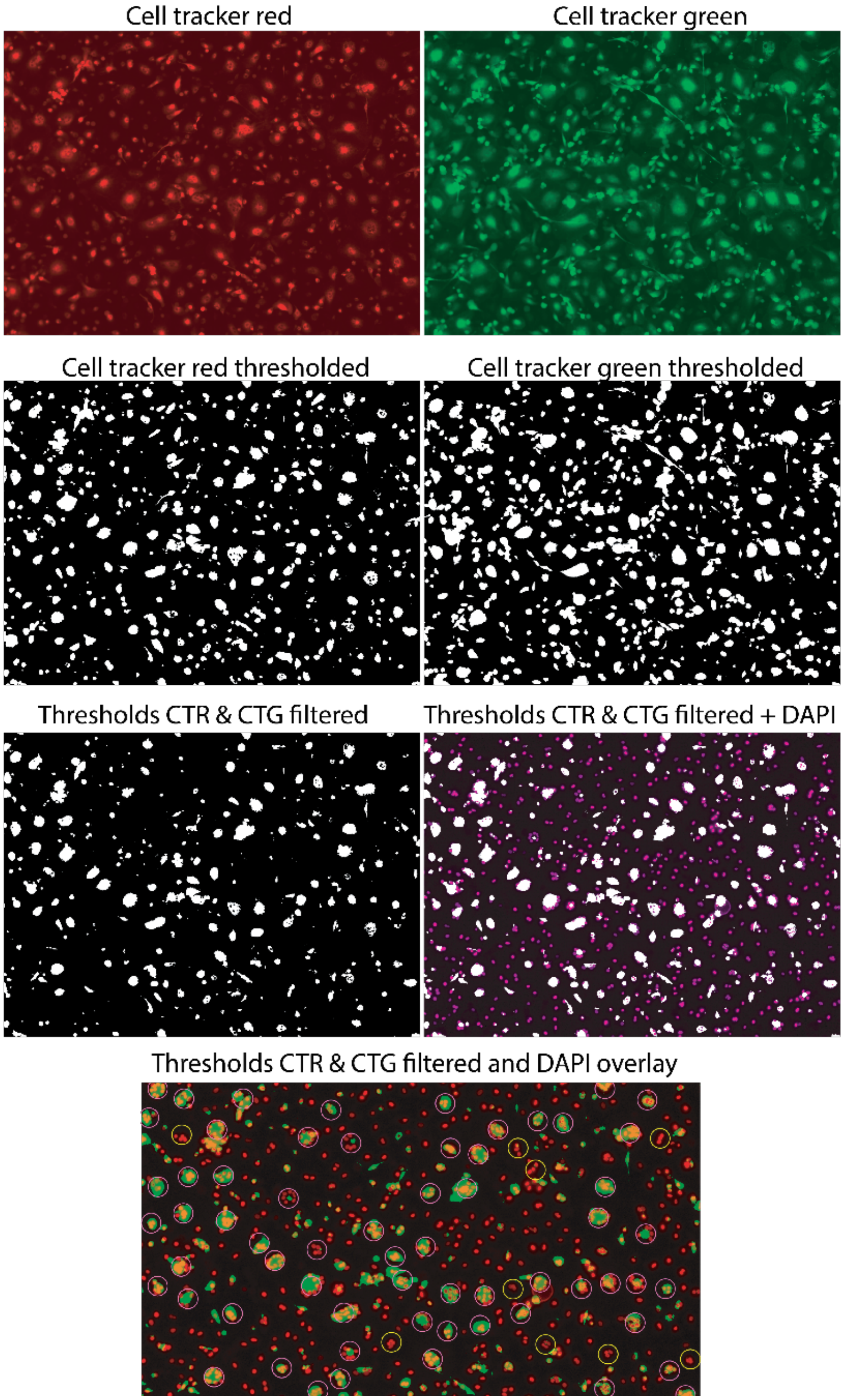

Figure S5. Example of macrophages from 2 pooled donors (D917, and D344) being able to fuse together. One donor was stained with cell tracker red, whilst the other was stained with cell tracker green. Subsequently, macrophages were cultured together and allowed to fuse for 2 days. Afterwards, imaging was performed in both channels, followed by thresholding. Next, a mask was made where the signals overlapped and the DAPI signal was added. In the bottom image, nuclei are stained in red, double positive cells in green, multinucleated cells from one donor are outlined with a yellow ring, and multinucleated cells from multiple donors are depicted with a purple ring.

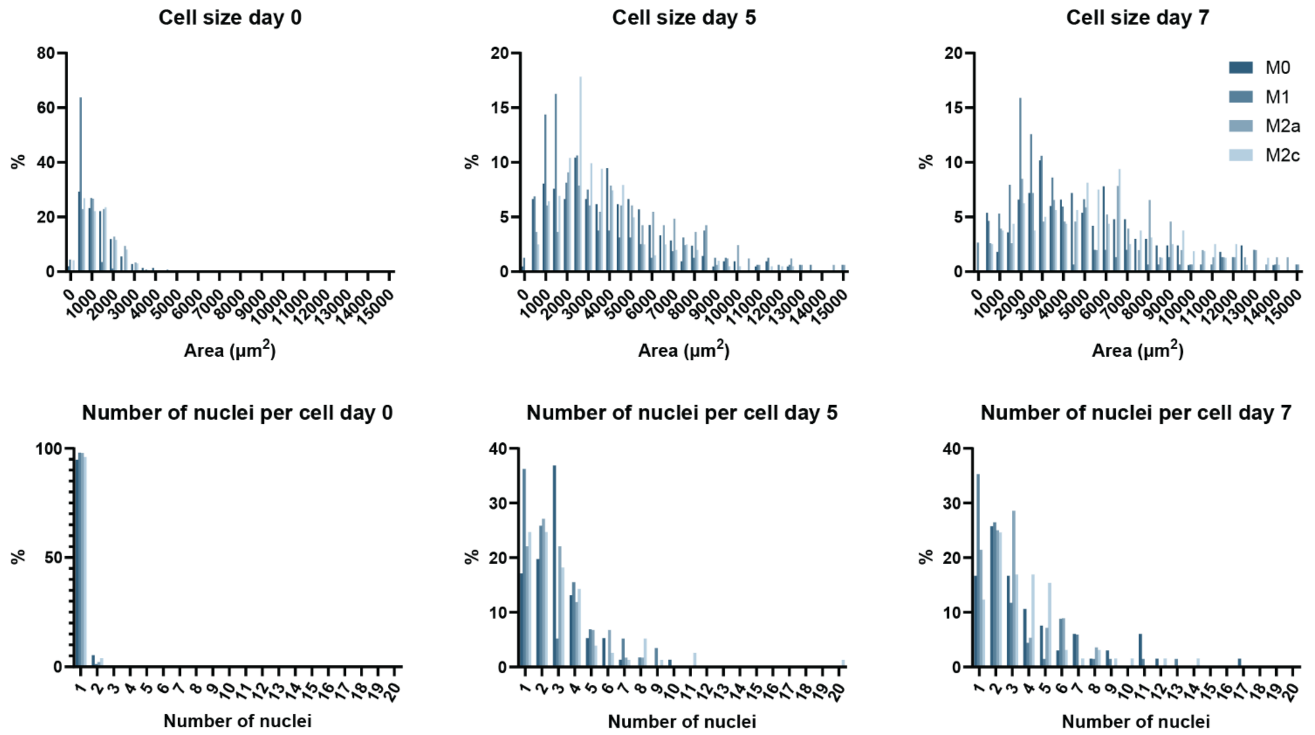

Figure S6. Cell size and number of nuclei per cell. Histogram view of cell size and number of nuclei per cell as seen in figure 7B in main manuscript. Note the higher percentages on the Y axis for the first graphs on the left.

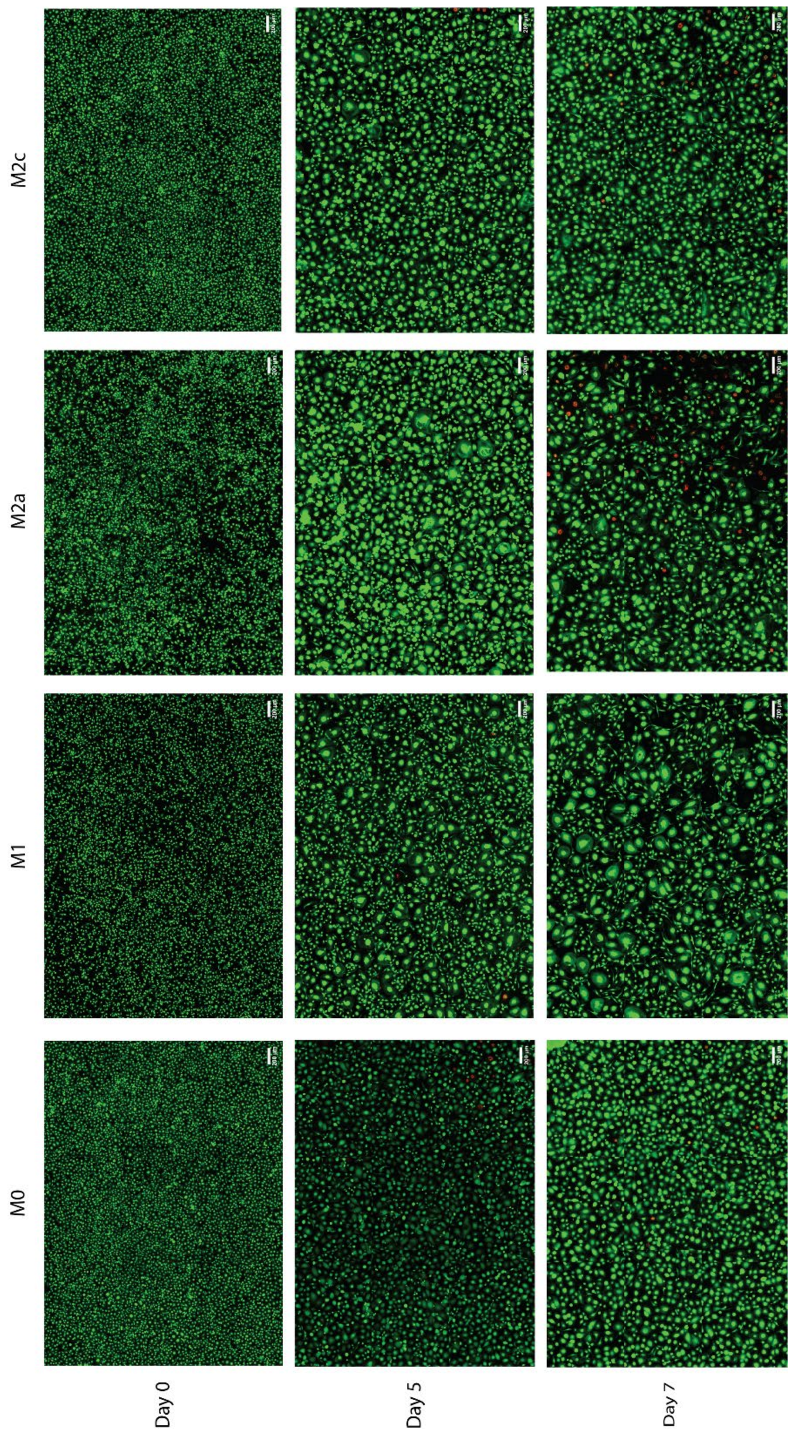

Figure S7. Live/Dead staining. Cells were incubated with Calcein AM (Green) and propidium Iodide (Red) for 5 minutes and subsequently imaged to investigate the viability of the cells. This was performed for all macrophage polarization states (M0, M1, M2a, M2c) and for all timepoints (Day 0; polarized macrophages, Day 5; FBGCs, Day 7; polarized FBGCs). Scalebar represents 200 µm.

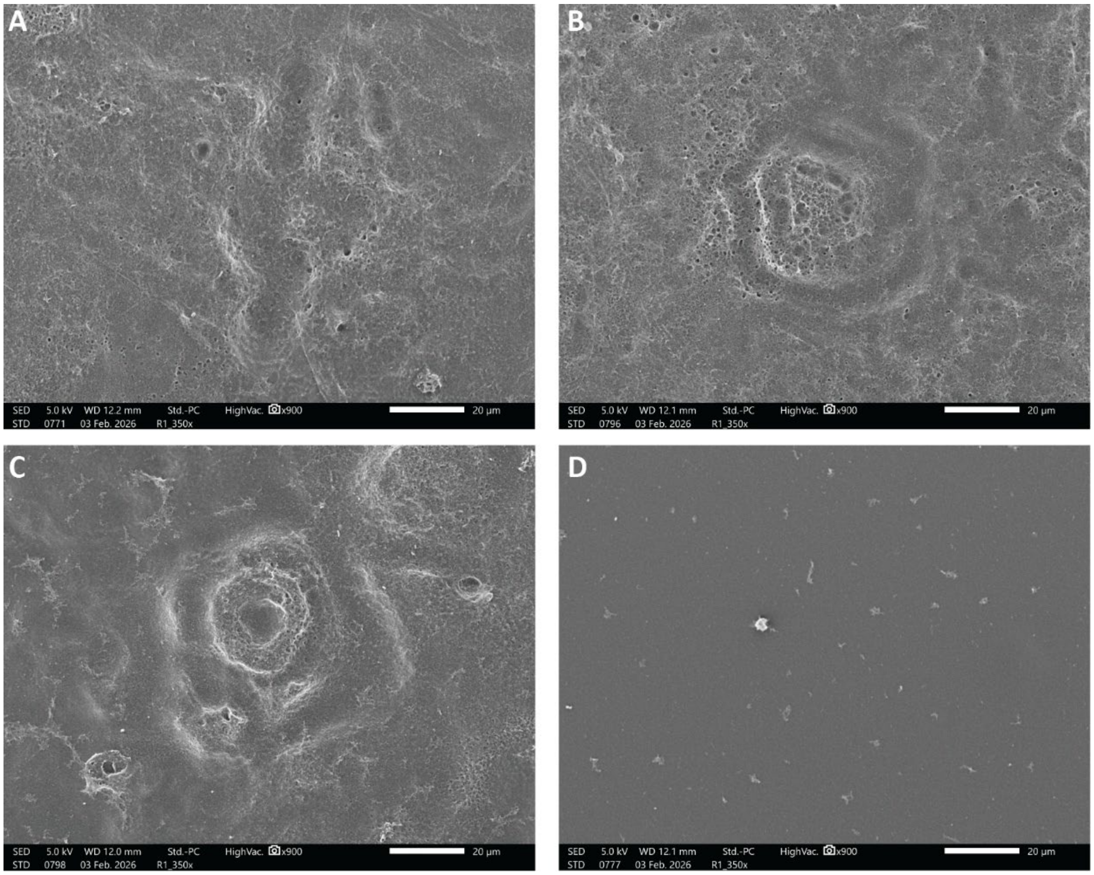

Figure S8. Confirmation of biomaterial degradation examples. Foreign body giant cells (FBGCs) (**A-C**) or fibroblasts (**D**) were cultured on polycaprolactone-bisurea (PCL-BU) following protocol B. Subsequently, cells were lysed, samples were dehydrated, and imaged using the JCM-7000 NeoScope™ (JEOL).
