# Supplementary material for: Macrophage polarization state influences fusion and phenotype of human foreign body giant cells in vitro
Source: Front Immunol. 2026 Jul 21;17:1832686. doi: 10.3389/fimmu.2026.1832686 (PMC13433290; doi:10.3389/fimmu.2026.1832686)
Supplement: Supplementary file 2 [file DataSheet2.pdf]

frontiers

The International Society for the Advancement of Cytometry (ISAC) has highlighted the importance of including comprehensive methodological information to ensure data reproducibility and reliability. In line with this, Frontiers in Immunology now requires authors to submit a checklist for manuscripts that involve flow or mass cytometry. This checklist helps standardize the reporting process, improving the quality and transparency of published data. By doing so, we support scientific progress, making it easier for other researchers to replicate and validate experiments.

This form should be submitted with any manuscripts using flow or mass cytometry.

Sample/specimen/material description

☐ Total blood

☑ PBMCs

☐ Organ digests

Other isolated macrophages

Did the samples suffer any treatment before or after incubation with the antibodies?

☑ Drug macrophages were stimulated with MCSF, followed by polarizing agents (LPS/IFNy, IL4, or IL10)

☐ Cell permeabilization ______________________

☐ Dye ______________________________________

☐ Propidium iodine

☐ Not applicable

Other blocking with trustain FcX (Biolegend)

Instrument and antibodies

Name of the Cytometer FACSCanto II; BD Biosciences

**Antibodies and targets**	**Fluorochrome/ Metal**	**Catalog number/Company**
e.g. anti-CD4	FITC	Cat. XXX/ XXX ltd.

anti-CD206	FITC	321118/BioLegend
anti-IL-4Ralpha	APC	355006/BioLegend
anti-IL-13Ralpha1	PE/Cy7	360408/BioLegend
anti-CD132 (common [illegible]	PE	338606/BioLegend
anti-CD11b	PerCP/Cy5.5	301328/BioLegend
anti-CD3	FITC	300306/BioLegend
anti-CD80	BV421	305221/BioLegend

Data analyses

1. Name of the software FlowJo
2. Reference gating strategy in the manuscript or supplementary material

Gating strategy in (eg Figure X) Figures S2 and S3
